# Exploring the Diversity and Systematics of *Phaeosphaeriaceae*: Taxonomic Novelties from Ecologically Diverse Habitats and Their Phylogenetic Resolution

**DOI:** 10.3390/jof9080853

**Published:** 2023-08-15

**Authors:** Dhanushka N. Wanasinghe, Sajeewa S. N. Maharachchikumbura

**Affiliations:** 1 Center for Mountain Futures, Kunming Institute of Botany, Chinese Academy of Sciences, Honghe 654400, China; dnadeeshan@gmail.com; 2 Center for Informational Biology, School of Life Science and Technology, University of Electronic Science and Technology of China, Chengdu 611731, China

**Keywords:** *Ascomycota*, multi-gene phylogeny, phragmosporous spores, taxonomy, terrestrial habitats

## Abstract

**Simple Summary:**

Our study describes several species of *Phaeosphaeriaceae* found in terrestrial habitats in Sichuan Province, China. We used morphological and molecular data to accurately delimit these species and determine their taxonomic relationships within the family. Our findings contribute to understanding fungal diversity in China and provide a basis for future studies exploring the ecological roles and biotechnological potential of these fungi. Additionally, our multi-gene phylogenetic approach provides increased accuracy and resolution in the delimitation of species boundaries within *Phaeosphaeriaceae*.

**Abstract:**

The family *Phaeosphaeriaceae* is a diverse group of ascomycetous fungi that are commonly found in terrestrial habitats worldwide. In recent years, there has been increasing interest in the biodiversity of Phaeosphaeriaceae in China, particularly in Sichuan Province, which has not been fully explored for its high fungal diversity. In our study, we conducted extensive surveys in Sichuan Province to identify and describe new species of *Ascomycota* with diverse habitats. Here, we present a taxonomic revision of *Phaeosphaeriaceae* with taxonomic novelties from terrestrial habitats in Sichuan Province based on a multi-gene phylogenetic approach. Our study focuses on the description of four new species of *Phaeosphaeriaceae*, representing novel additions to the diversity of this fungal family. Using a combination of morphological and molecular data, we provide detailed descriptions of the new taxa and their placement within the family. Additionally, we discuss the phylogenetic relationships of these new taxa with other members of *Phaeosphaeriaceae*, providing insights into the correct taxonomic classification of the family. Our taxonomic revision contributes to understanding fungal diversity in China and provides a foundation for future studies investigating the taxonomy and ecological roles of *Phaeosphaeriaceae* fungi. Furthermore, our multi-gene phylogenetic approach provides increased resolution and accuracy in the delimitation of species boundaries within the family. Our study highlights the importance of continued exploration and taxonomic revision in order to fully understand the diversity and distribution of fungal species in China and beyond. **New species:** *Paraloratospora sichuanensis*, *Phaeosphaeria chengduensis*, *P. sichuanensis*, and *Septoriella shoemakeri*. **New combinations:** *Paraloratospora breonadiae*, *P. fructigena*, *Septoriella ammophilicola*, *S. asparagicola*, *S. festucae*, *S. luzulae*, and *S. verrucispora*. **New names:** *Septoriella paradactylidis*, and *S. neomuriformis*.

## 1. Introduction

The family *Phaeosphaeriaceae* comprises a diverse group of ascomycetous fungi that are commonly found in terrestrial habitats worldwide [1]. Despite their abundance, the taxonomy of *Phaeosphaeriaceae* remains obscure, with many species remaining undescribed or poorly characterized. In recent years, there has been an increasing interest in the biodiversity of *Phaeosphaeriaceae*. Researchers have conducted extensive surveys of the mycobiota, resulting in the discovery of over 135 new taxa in *Phaeosphaeriaceae* within the last five years: 2019–2023 [2]. In particular, several new species of *Phaeosphaeria* have been identified, representing novel additions to the diversity of this fungal family. However, the taxonomy of *Phaeosphaeriaceae* is still challenging due to the high degree of morphological variability within the family. Many species have been described based on only a single specimen, and the use of molecular data to aid in the delimitation of species boundaries for the species published prior to the 1990s is still inadequate. Currently, the family consists of 84 genera [3], but their exact classification is debatable. The phylogenetic relationships within the family are also not well resolved. Twenty-one of the genera are represented by a single strain in the family (viz., *Acericola*, *Alloneottiosporina*, *Arezzomyces*, *Banksiophoma*, *Bhagirathimyces*, *Bhatiellae*, *Camarosporioides*, *Equiseticola*, *Hydeopsis*, *Jeremyomyces*, *Kwanghwaensis*, *Longispora*, *Melnikia*, *Neosphaerellopsis*, *Ophiosimulans*, *Pseudophaeosphaeria*, *Scolicosporium*, *Vittaliana*, *Vrystaatia*, *Xenophaeosphaeria*, and *Xenophoma*), whereas thirty of the genera are represented by a single species (monotypic). Additionally, some of the species are not monophyletic with their type (i.e., *Muriphaeosphaeria*, *Loratospora*, and *Phaeosphaeria*). Since the last revision [4], there has been no proper attempt to resolve the inter- and intra-generic classification of *Phaeosphaeriaceae*, indicating the need for a new taxonomic revision of the family.

Collecting species in *Phaeosphaeriaceae* is crucial because it enables more accurate and precise identification of its species, which is particularly important for linking their holomorphs and finding DNA-based sequence data for old herbarium specimens. Moreover, this helps fill the gap in our understanding of their diversity and evolution [5]. Since *Phaeosphaeriaceae* are cosmopolitan and ecologically important, studying their ecological relationships is essential to understand them in a broader sense [1,4]. In addition, some genera (i.e., *Polydomus* and *Setophoma*) have significant practical applications, including as sources of biological activities and biotechnological products [6,7,8]. Therefore, conducting research on *Phaeosphaeriaceae* is an important step towards unlocking their potential applications. Accurate identification of *Phaeosphaeriaceae* species is also necessary for assessing their conservation status and identifying areas of high biodiversity that require protection.

This paper presents a taxonomic revision of *Phaeosphaeriaceae*, focusing on the genera *Paraloratospora*, *Phaeosphaeria*, and *Septoriella*. Four new species, seven new combinations, and two new names are proposed, supported by detailed morphological and molecular analyses to establish their placement within this family. Furthermore, we investigate the phylogenetic relationships of these novel taxa with other members of *Phaeosphaeriaceae* and their current taxonomic classification. Additionally, we confirm the species list that includes DNA data in *Phaeosphaeria* sensu stricto. This study highlights the significance of ongoing exploration and taxonomic revision to comprehensively understand fungal species diversity and distribution in China and beyond. It contributes to our knowledge of the diversity and evolution of *Phaeosphaeriaceae* in terrestrial habitats and establishes a foundation for future taxonomic and ecological investigations within this important fungal family.

## 2. Materials and Methods

### 2.1. Isolates and Specimens

During our fieldwork in Sichuan Province, China, we collected typical black ascomata/conidiomata from dead twigs across various regions including Chenghua District, Qingyang District, and Dayi County during both wet (August 2021) and dry (January 2023) seasons. The collected samples were placed in Ziploc bags and transported to the mycology laboratory of the Kunming Institute of Botany, where they were stored in paper envelopes. Single spore isolation was conducted according to the methods described in Wanasinghe et al. [9], and germinated spores were handled appropriately. Dried specimens were preserved in the fungarium of the Cryptogams Kunming Institute of Botany, Academia Sinica (KUN-HKAS), and representative cultures were deposited in the Kunming Institute of Botany Culture Collection (KUMCC) and the University of Electronic Science and Technology Culture Collection (UESTCC), Chengdu, China. Nomenclatural data for fungal novelties were deposited in MycoBank [10].

### 2.2. Morphological Observations

To examine the ascomata, conidiophores, and conidia from natural substrates, we rehydrated them with tap water and viewed them using a Motic SMZ 168 series stereo-microscope (Motic Asia, Kowloon, Hong Kong). Morphological characteristics were evaluated by hand-sectioning sporocarps placed on water-mounted glass slides. We assessed various characteristics such as the diameter, height, color, and shape of the ascomata/conidiomata, as well as the width of the peridium and the height and diameter of the ostioles. Microscopic photography was carried out using a Nikon ECLIPSE Ni (Nikon Corporation, Konan, Minato-ku, Tokyo, Japan) compound microscope with differential interference contrast (DIC) and phase contrast (PC) illumination. Images of microscopic structures were captured using a Canon EOS 600D (Canon Inc., Ota, Tokyo, Japan) camera. Additionally, macroscopic images of colonies were documented using an iPhone XS Max (Apple Inc., Cupertino, CA, USA) in daylight. We used the Tarosoft (R) Image Frame Work program to measure dimensions, and images used for figures were processed using Adobe Photoshop CS6 (Adobe Systems, San Jose, CA, USA).

### 2.3. DNA Extraction, PCR Amplifications, and Sequencing

To extract genomic DNA, we followed the methods described by Wanasinghe et al. [11] using the Biospin Fungus Genomic DNA Extraction Kit-BSC14M1 (BIOER, Hangzhou Bioer Technology Co., Ltd., Hangzhou, China) according to the manufacturer’s instructions. Reference DNA for the polymerase chain reaction (PCR) was stored at 4 °C for regular use and duplicated at −20 °C for long-term storage. We used primers ITS5/ITS4, LR0R/LR5, NS1/NS4, EF1-983F/EF1-2218R, and fRPB2-5f/fRPB2-7cR to amplify the DNA sequences of the internal transcribed spacers (ITS), partial 28S large subunit rDNA (LSU), partial 18S small subunit rDNA (SSU), translation elongation factor 1-α (*tef*1), and RNA polymerase II second largest subunit (*rpb*2) [12,13,14,15,16,17]. The protocols used for PCR amplification (SSU, LSU, ITS, *tef*1, and *rpb*2) were the same as those described in Wanasinghe and Mortimer [18]. The amplified PCR fragments were sent to a private company (BGI, Ltd., Shenzhen, China) for sequencing.

### 2.4. Molecular Phylogenetic Analyses

#### 2.4.1. Sequencing and Sequence Alignment

To analyze the sequences generated from different primers of the five genes, we conducted a BLAST search to identify sequences with high similarity indices and find the closest matches with taxa in *Phaeosphaeriaceae*, following recent publications [19,20,21,22]. Later, we analyzed them with other sequences retrieved from GenBank (Table 1). We used MAFFT v. 7 [23] to automatically generate multiple alignments of all consensus sequences as well as the reference sequences. We manually corrected the alignments using BioEdit v. 7.0.5.2 [24] where necessary.

#### 2.4.2. Phylogenetic Analyses

We examined the single-locus datasets for any topological incongruence among the loci for members of the analyses. The resulting alignments were then concatenated into a multi-locus alignment and analyzed using maximum likelihood (ML) and Bayesian (BI) phylogenetic methods in the CIPRES Science Gateway [25]. We obtained an ML tree using RAxML-HPC2 on XSEDE v. 8.2.10 [26] with a GTR + G + I model and calculated support values with 1000 bp replicates [27]. Nodes with ML bootstrap values equal to or greater than 75% are indicated above each node.

The best-fit model was selected based on Bayesian Information Criterion (BIC) scores using the IQ-TREE web application at http://iqtree.cibiv.univie.ac.at (accessed on 27 June 2023) [28]. We restricted the pool of available models to JC, F81, HKY, SYM, and GTR for model selection. We performed BI with two parallel runs of 50 M generations, using four chains in each, and retaining one tree every 100 generations. The dataset was partitioned by gene, and HKY + I + G (SSU), SYM + I + G (LSU), and GTR + I + G (ITS, *tef*1, and *rpb*2) models were applied to each partition, ending the run automatically when the standard deviation of split frequencies dropped below 0.01 with a burn-in fraction of 0.25. A 50% majority rule consensus tree was obtained after discarding the first 25% of trees, and posterior probabilities were used as a measure of nodal support. Nodes with posterior probabilities in BI (BYPP) greater than 0.95 are indicated above each node. Finally, we visualized the phylograms using the FigTree v1.4.0 program [29] and reorganized them in Microsoft PowerPoint (2019).

## 3. Results

### 3.1. Phylogenetic Analyses

**Figure 1:**
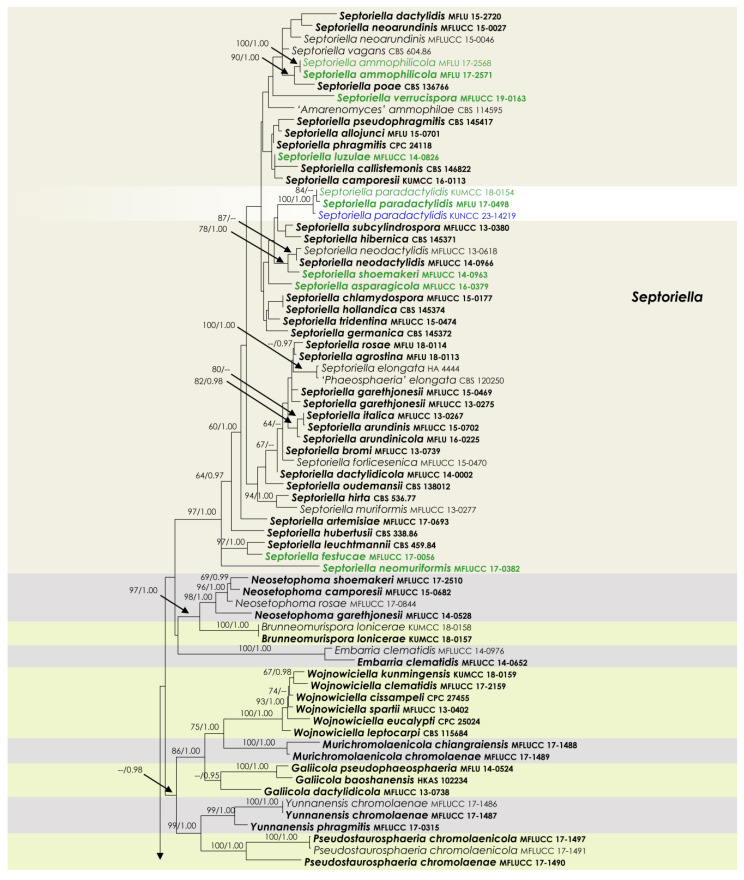

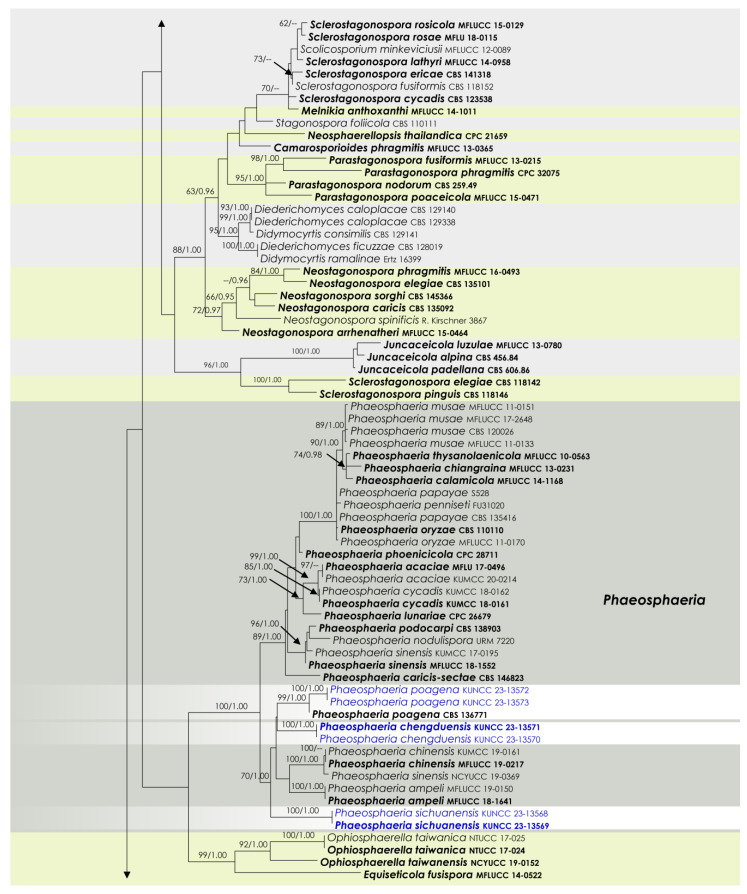

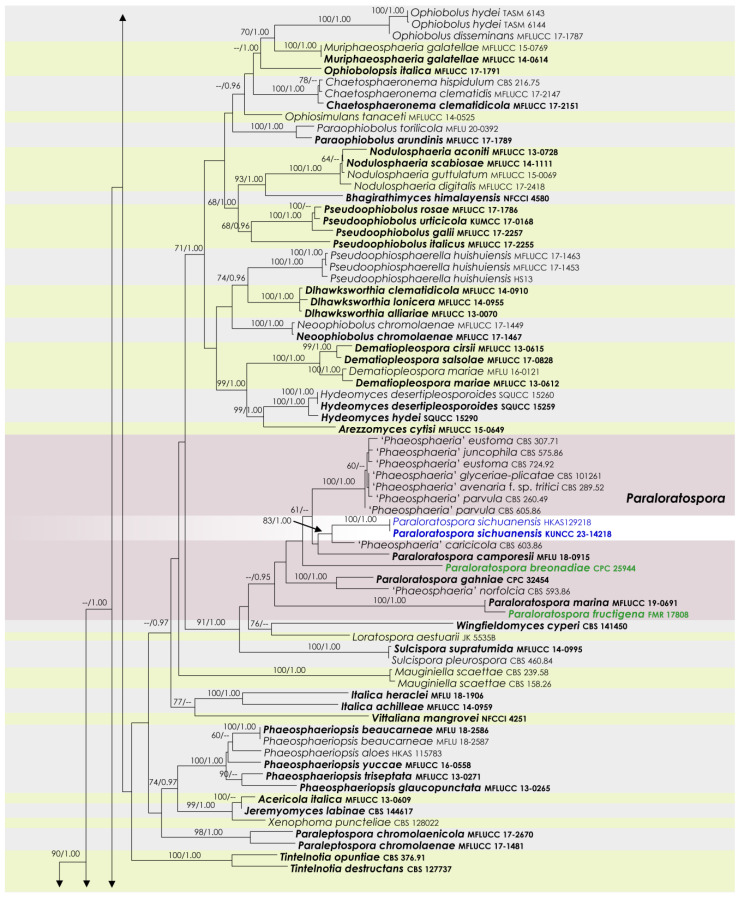

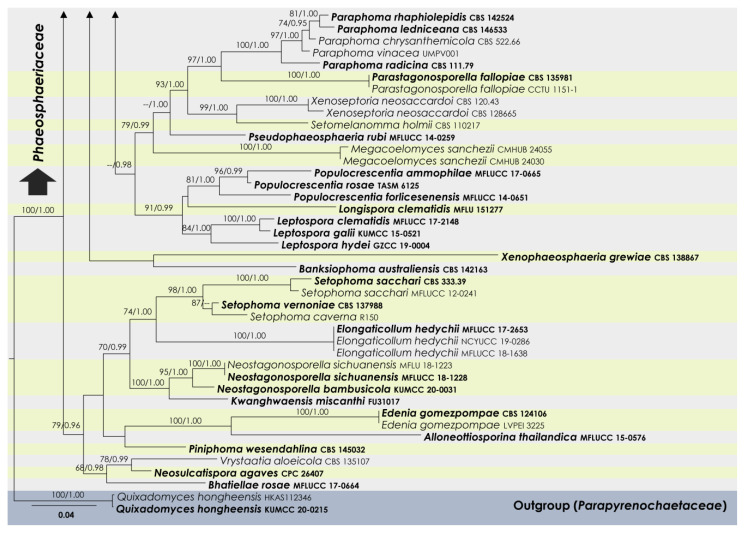
RAxML tree based on a combined dataset of partial SSU, LSU, ITS, *tef*1, and *rpb*2 DNA sequence analyses in *Phaeosphaeriaceae*. The bootstrap support values for ML (MLB) greater than or equal to 70% and Bayesian posterior probabilities (BYPP) greater than or equal to 0.95 are indicated as MLB/BYPP above the corresponding nodes. The newly analyzed isolates are highlighted in blue. The scale bar in the figure represents the expected number of nucleotide substitutions per site.

To examine the evolutionary relationships of our new strains within *Phaeosphaeriaceae*, we conducted phylogenetic analyses based on the combined SSU, LSU, ITS, *tef*1, and *rpb*2 DNA sequences of 258 representatives of the family. We used two strains from *Quixadomyces hongheensis* (*Parapyrenochaetaceae*) as the outgroup taxon. The full dataset consisted of 4413 characters, including gaps (SSU = 999 characters, LSU = 846, ITS1 + 5.8S + ITS2 = 663, *tef*1 = 871, and *rpb*2 = 1034). The RAxML analysis of the combined dataset yielded a best-scoring tree with a final ML optimization likelihood value of −60,293.418721. The matrix had 2211 distinct alignment patterns, with 39.5% undetermined characters or gaps. We used the GTR + I + G model of the combined amplicons for the analysis, with the following parameters: estimated base frequencies of A = 0.245652, C = 0.237255, G = 0.265783, and T = 0.251310; substitution rates of AC = 1.259144, AG = 3.770291, AT = 2.003190, CG = 0.806828, CT = 7.047351, and GT = 1.000; proportion of invariable sites I = 0.575954; and gamma distribution shape parameter α = 0.641179. The Bayesian analysis ran 40,280,000 generations before the average standard deviation for split frequencies reached below 0.01 (0.009998). The analyses generated 40,281 trees, from which we sampled 30,211 trees after discarding the first 25% as burn-in. The alignment contained a total of 2217 (SSU: 340, LSU: 325, ITS: 461, *tef*1: 415, and *rpb*2: 676) unique site patterns. Where applicable, the phylogenetic results obtained (Figure 1) are discussed in the descriptive notes below.

### 3.2. Taxonomy

***Pleosporales*** Luttr. ex M.E. Barr, Prodromus to class Loculoascomycetes: 67 (1987).

***Phaeosphaeriaceae*** M.E. Barr, Mycologia 71: 948 (1979).

***Paraloratospora*** Bundhun, Tennakoon, Phookamsak & K.D. Hyde, Fungal Diversity 100: 101 (2020).

Notes: This study presents an updated and comprehensive phylogenetic classification of the genus *Paraloratospora*, incorporating SSU, LSU, ITS, *tef*1, and *rpb*2 DNA sequence analyses. By combining morphological and phylogenetic considerations, we have identified a new species, *Paraloratospora sichuanensis*, as well as proposed two new combinations, *Paraloratospora breonadiae* (=*Phaeosphaeria breonadiae*) and *P. fructigena* (=*Phaeosphaeria fructigena*), within the genus. Detailed information regarding these taxonomic changes can be found in the note sections, where an additional discussion and supporting evidence are provided. The discovery of *Paraloratospora sichuanensis* adds to the known diversity of *Paraloratospora* species and expands our knowledge of the ecological and morphological characteristics within the genus. The two newly proposed combinations, *Paraloratospora breonadiae* and *P. fructigena*, reflect the revised taxonomic understanding based on the integration of molecular and morphological data. These taxonomic changes contribute to the overall refinement and accuracy of the classification system for *Paraloratospora*.

***Paraloratosporabreonadiae*** (Crous & Jol. Roux) Maharachch. & Wanas. **comb. nov.**

MycoBank: MB 849359.

*≡Phaeosphaeria breonadiae* Crous & Jol. Roux, Persoonia 36: 399 (2016).

Holotype: South Africa, Limpopo Province, Wolkberg, on leaves of *Breonadia microcephala*, January 2015, J. Roux (CBS H-22631, holotype), ex-type, CPC 25944, CBS 141334.

Descriptions and illustrations: See Crous et al. [30].

Notes: The phylogenetic analysis conducted in this study yielded interesting results regarding the relationship between *Phaeosphaeria breonadiae* and *P. fructigena* with the species of *Paraloratospora*. The analysis revealed that type strains of these two species clustered together with *Paraloratospora*, forming a distinct group separate from the *Phaeosphaeria* sensu stricto (Figure 1). Based on these findings, it was determined that a reclassification of these two species within the genus *Paraloratospora* was warranted. Consequently, we propose the new combinations *Paraloratospora breonadiae* comb. nov. and *P. fructigena* comb. nov. for *Phaeosphaeria breonadiae* and *P. fructigena*, respectively. This reclassification aligns with the phylogenetic evidence and provides a more accurate taxonomic placement for these species within the *Paraloratospora* genus. The newly proposed combinations reflect the closer relationship and shared characteristics observed between these species and other members of *Paraloratospora*.

**Figure 2:**
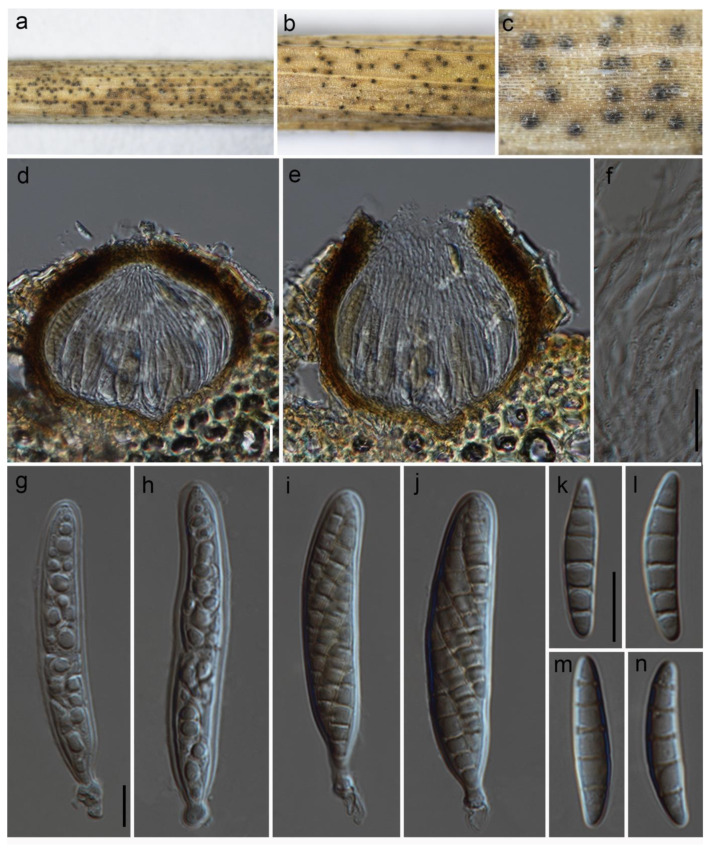
***Paraloratospora sichuanensis*** (HKAS 129217, holotype): (**a**–**c**) ascomata on the host; (**d**,**e**) cross section of ascomata; (**f**) pseudoparaphyses; (**g**–**j**) asci; (**j**–**n**) ascospores. Scale bars: (**d**,**f**) 20 µm; (**g**,**k**) 10 µm (scale bar of (**d**) applies to (**e**), scale bar of (**g**) applies to (**g**–**j**), and scale bar of (**k**) applies to (**k**–**n**)).

***Paraloratospora sichuanensis*** Maharachch. & Wanas. **sp. nov.** (Figure 2).

MycoBank: MB 849432.

*Etymology*: The specific epithet is derived from Sichuan, where this fungus is collected.

Holotype: HKAS 129217

The fungus is a *saprobe*, found on dead clumps of *Lolium perenne* L. (*Poaceae*). In its sexual morph, *Ascomata* are 130–190 μm high and have a 120–210 μm diam. (M = 165 × 170 µm, n = 10), are semi-immersed to erumpent, solitary, scattered, and uniloculate, and can be globose to subglobose in shape, with a brown to dark brown color. The *ostioles*, which can reach up to 3–40 µm in diam., are centrally located and have a minute papilla. They are dark brown and consist of hyaline periphyses. The *peridium* is of unequal thickness, with a thicker portion near the apex (15–24 µm) and a thinner portion at the base (10–16 µm). It is composed of two layers, with the outer part consisting of dark brown thick-walled cells arranged in a textura angularis pattern and the inner layer made up of pale brown to hyaline thin-walled cells also arranged in a textura angularis pattern. The *hamathecium* is 2.5–4.5 µm wide, unbranched, septate, and composed of pseudoparaphyses that are constricted at the septum. The *asci* 55–90 × 7–14 µm (M = 62.3 × 11 µm, n = 15) are eight-spored, bitunicate, fissitunicate, cylindrical, sessile to subsessile, with a rounded apex and a thick wall. They are short pedicellate and possess a developed ocular chamber. The *ascospores* 20–28 × 3–5.5 µm (M = 23 × 4.5 µm, n = 25) are arranged in overlapping 1–2 seriate fashion and are 3–5 septate (mostly 5). The ascospores are hyaline to pale yellowish in color and have a fusiform shape with rounded ends. They can be straight to slightly curved and have a smooth surface. They are surrounded by a sheath. Asexual morph: the asexual form of this fungus is undetermined.

Culture characteristics: The ascospores germinated on PDA within 24 h. Following a two-week incubation period at 25 °C, the colonies on PDA medium reached a diameter of 5 cm. These colonies exhibited an undulate margin, initially appearing creamy whitish and transitioning to a creamy grey shade at the center. Towards the periphery, the color lightened further. The colonies were slightly raised in texture, with a creamy orange hue at the center and a creamy grey coloration towards the periphery when observed from the reverse side.

Known distribution: China (Sichuan) on *Lolium perenne* in terrestrial habitats.

Material examined: China, Sichuan, Chengdu, Chenghua District, The Chengdu Research Base of Giant Panda Breeding, on dead culms of *Lolium perenne*, 13 August 2021, Q Wang, W90-4-3 (HKAS 129217, holotype), ex-type culture, KUNCC 23-14218. ibid. W90-4-1 (HKAS 129218).

Notes: Based on the phylogenetic analysis of multiple genes, *Paraloratospora sichuanensis* shares a close relationship with a putatively named strain called ‘*Phaeosphaeria*’ *caricicola* (CBS 603.86). However, it is important to note that de Gruyter et al. [31] provided sequence data for CBS 603.86 without establishing a connection to any type material. In terms of morphology, *Paraloratospora sichuanensis* does not significantly differ from *Phaeosphaeria caricicola* in several aspects. Both species exhibit similar dimensions in terms of ascomata size (130–190 μm high, 120–210 μm diameter for *P. sichuanensis*, compared to 120–140 μm high, 120–140 μm diameter for *Phaeosphaeria caricicola*), asci size (55–90 × 7–14 µm for *P. sichuanensis*, compared to 60–70 × 6–8 µm for *Phaeosphaeria caricicola*), and ascospore size (20–28 × 3–5.5 µm for *P. sichuanensis*, compared to 15–20 × 3.5–4 µm for *Phaeosphaeria caricicola*) [32,33]. However, a notable difference lies in the number of septa observed in the ascospores of these two species. The ascospores of *P. sichuanensis* predominantly possess five septa, whereas *Phaeosphaeria caricicola* typically has ascospores with four septa [33]. It is worth noting that further investigation is required to establish the exact phylogenetic placement of the type of *Phaeosphaeria caricicola*.

***Paraloratospora fructigena*** (Magaña-Dueñas, Cano-Lira & Stchigel) Maharachch. & Wanas. **comb. nov.**

MycoBank: MB 849360.

≡*Phaeosphaeria fructigena* Magaña-Dueñas, Cano-Lira & Stchigel, Journal of Fungi 7 (12, no. 1102): 11 (2021).

Holotype: Spain, Tarragona Province, Capafonts (41.29598, 1.02753), from freshwater submerged plant debris, March 2019, V Magaña-Dueñas and II González (CBS H-24910), ex-type FMR 17808, CBS 148658.

Descriptions and illustrations: See Magaña-Dueñas et al. [34].

Notes: *Phaeosphaeria fructigena*, which was isolated from plant debris submerged in freshwater, is characterized by the production of clavate asci and fusiform ascospores. The sexual stage of *P. fructigena* has only been observed in both the original material and pure cultures by Magaña-Dueñas et al. [34]. In our phylogenetic analysis, *P. fructigena* was grouped under *Paraloratospora*. Please refer to the notes under *Paraloratospora breonadiae* for further information.

***Phaeosphaeria*** I. Miyake, Bot. Mag. (Tokyo) 23: 93 (1909).

Notes: The genus *Phaeosphaeria* is renowned for its significant diversity, encompassing numerous described species and many more yet to be discovered. The presence of *Phaeosphaeria* species has been reported in various ecosystems, including terrestrial and freshwater environments [34,35]. These fungi exhibit adaptability to a wide range of climatic conditions and substrates, allowing their distribution in both temperate and tropical regions. Within this genus, some species play a crucial role as saprophytes, participating in vital processes such as decomposition and nutrient cycling. They are commonly associated with decaying plant material, dead wood, or organic debris of monocotyledons. On the other hand, certain *Phaeosphaeria* species are recognized as plant pathogens, causing diseases in various hosts, including agricultural crops and forest trees [4,35].

In our study, we observed that six strains of our newly isolated species exhibit close resemblance to *Phaeosphaeria* strains based on BLAST similarity indices in GenBank. These findings underscore the importance of conducting further investigations and taxonomic revisions to ensure accurate classification and a better understanding of the relationships within the *Phaeosphaeria* genus. By utilizing a combination of morphological and molecular data from these new collections, we provide detailed descriptions of two new species within *Phaeosphaeria*. Additionally, we revised the species description for *Phaeosphaeria poagena* to update the characteristics of its sexual form.

**Figure 3:**
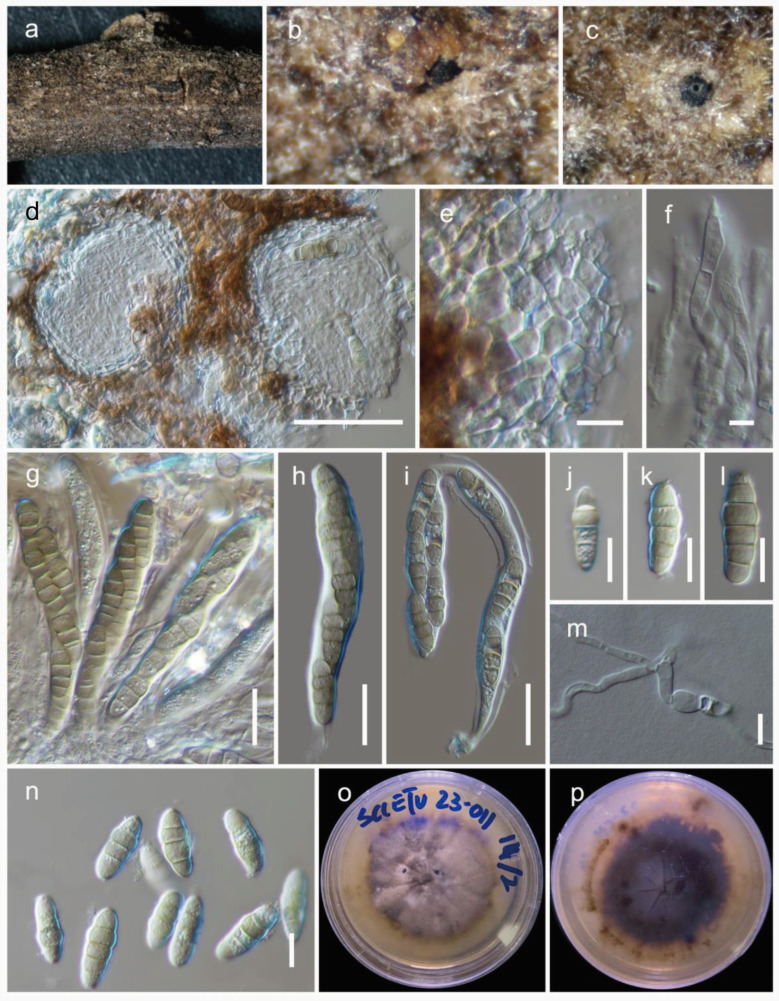
***Phaeosphaeria chengduensis*** (HKAS 129197, holotype): (**a**–**c**) herbarium specimen; (**d**) vertical section through ascomata; (**e**) peridium; (**f**) pseudoparaphyses; (**g**–**i**) asci; (**j**–**n**) ascospores (m germinated spore); (**o**,**p**) colonies on PDA after 6 weeks. Scale bars: (**d**) 50 µm; (**e**,**j**–**n**) 10 µm; (**f**) 5 µm; (**g**–**i**) 20 µm.

***Phaeosphaeria chengduensis*** Wanas. & Maharachch. **sp. nov.** (Figure 3).

MycoBank: MB 849352.

*Etymology*: The specific epithet is derived from Chengdu, where this fungus was collected.

Holotype: HKAS 129197.

The fungus is *saprobic*, found on a dead twig of an unidentified deciduous host. Sexual state: The *ascomata* are 100–170 μm high, 90–150 μm in diam., and mostly scattered and immersed, with a uniloculate structure that is globose to subglobose and brown to dark brown in color. The *peridium* is 10–20 μm wide and consists of two layers: an outer layer composed of brown to dark brown cells arranged in a textura angularis pattern and an inner layer with loosely arranged, hyaline cells in a textura angularis to textura globulosa pattern. The *hamathecium* is made up of pseudoparaphyses, measuring 2–3 μm in width, which are cellular and indistinctly constricted at the septa. The *asci* measure 80–100 × 12–15 μm (M = 90.6 × 13.6 μm, n = 15) and are eight-spored, bitunicate, fissitunicate, broadly cylindrical, and have a pedicel. They are rounded at the apex and possess an ocular chamber. *Ascospores* measure 18–26 × 6.5–9 μm (M = 20.6 × 7.8 μm, n = 20) and are arranged as overlapping 1–2 seriate. They are phragmosporous, ellipsoidal with rounded ends, and initially yellowish brown but becoming pale brown as they mature. The *ascospores* are 4–5 septate and exhibit slight constriction at the septa, with the upper cell next to the middle septum being enlarged. Their surfaces are smooth walled. The asexual state was not observed.

Culture characteristics: Colonies on PDA reach a diameter of up to 3 cm after four weeks at 25 °C. These colonies have an irregular, flattened to slightly raised appearance, with a greenish-grey edge and a grey center. They also display various color sectors ranging from grey to dark grey. The reverse side of the colonies appears dark brown.

Known distribution: China (Sichuan) on dead twigs of deciduous hosts in terrestrial habitats.

Material examined: China, Sichuan, Chengdu, Chenghua, 30.748056 N, 103.928889 E, 533 m, on dead twigs of an unknown deciduous host, 03 January 2023, D.N. Wanasinghe, SCCETU23-011-4 (HKAS 129197, holotype), ex-holotype culture, KUNCC 23-13571. ibid. 30.747694 N, 103.928749 E, 03 January 2023, SCCETU23-011-1 (HKAS 129198), living culture, KUNCC 23-13570.

Notes: Within *Phaeosphaeria*, our novel fungus closely resembles *P. arenaria* (≡*Leptosphaena arenaria*) and *P. hiemalis* (≡*Leptosphaeria hiemalis*) based on the presence of 4–5 septate, pigmented, ellipsoidal ascospores with rounded ends [33]. *Phaeosphaeria hiemalis* was collected from *Equisetum hyemale* (*Equisetaceae*) in Canada and USA. *Phaeosphaeria arenaria* was collected from *Festuca arenaria* (Poaceae) and *Phleum arenarium* (Poaceae), although the exact location is unknown [33]. Both of these species lack DNA-based sequence data for molecular comparisons. In our phylogenetic analysis, *Phaeosphaeria chengduensis* shows a close phylogenetic affinity to *P. poagena*. Morphologically, *Phaeosphaeria poagena* produces conical, fusiform spores with 3 septa, whereas *P. chengduensis* has ellipsoidal spores with rounded ends and 4–5 septa. However, their affiliation is not statistically supported, with a greater than or equal to 70% MLB or 0.95 BYPP. The comparison of nucleotide differences of ITS, *tef*1, and *rpb*2 between *Phaeosphaeria chengduensis* and *P. poagena* were 17/492 (3.4%), 25/869 (2.9%), and 64/621 (10.3%), respectively.

**Figure 4:**
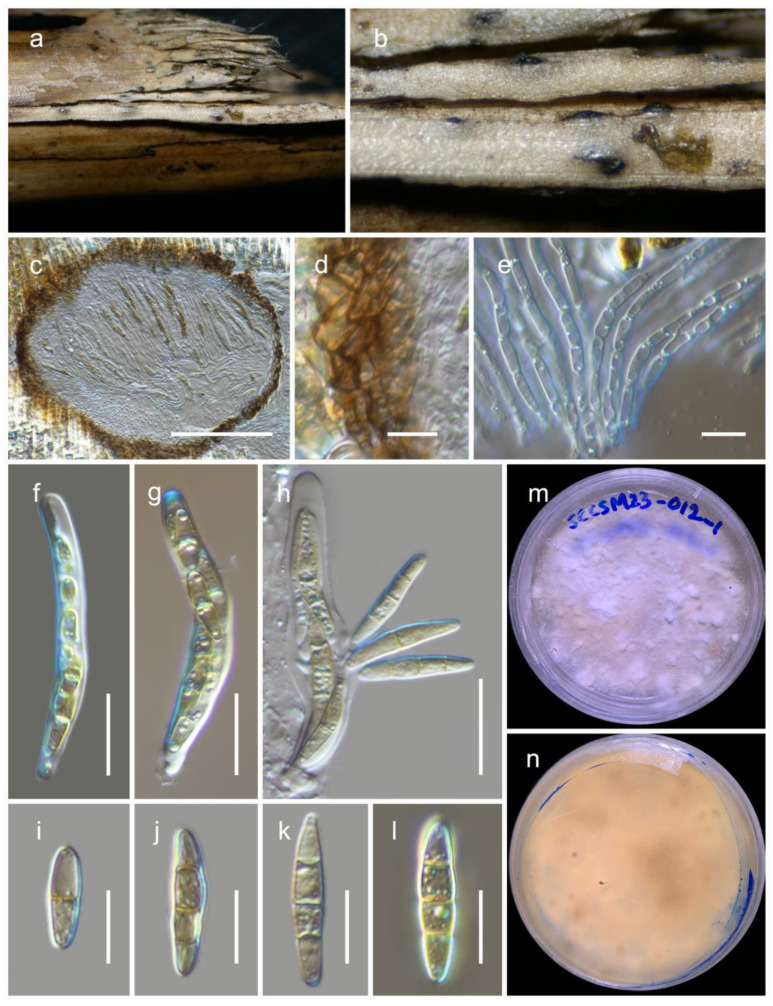
***Phaeosphaeria poagena*** (HKAS 129196): (**a**,**b**) ascomata on the bamboo culms; (**c**) vertical section through an ascoma; (**d**) peridium; (**e**) pseudoparaphyses; (**f**–**h**) asci ((**h**) showing a fissitunicate ascus); (**i**–**l**) ascospores; (**m**,**n**) colonies on PDA after 6 weeks. Scale bars: (**c**) 100 µm; (**d**,**e**,**i**–**l**) 10 µm; (**f**–**h**) 20 µm.

***Phaeosphaeria poagena*** Crous & Quaedvlieg. Persoonia 32: 195 (2014) ***amend*** (Figure 4).

MycoBank: MB 808889.

The fungus is *saprobic*, found on deceased bamboo (*Poaceae*). Sexual state: *Ascomata* are 200–250 μm high, 240–280 μm in diam., and mostly scattered. They are typically scattered, partially embedded, or semi-erupted, appearing as small black dots on the surface of the host. These *ascomata* are unilocular, globose to subglobose, and range in color from brown to dark brown. The *peridium*, 15–25 μm wide, consists of thin-walled cells arranged in a textura angularis pattern, and is composed of brown to dark brown cells. The *hamathecium* is made up of numerous pseudoparaphyses, measuring 2–3 μm in width, that are cellular and contain guttulate material. These *pseudoparaphyses* are constricted at the septa. The *asci* measure 65–80 × 8.5–10.5 μm (M = 71.6 × 9.8 μm, n = 15), are eight-spored, bitunicate, fissitunicate, broadly cylindrical, and have a short pedicel. They are rounded at the apex and possess an ocular chamber. *Ascospores* measure 18–26 × 4–5 μm (M = 23.2 × 4.6 μm, n = 20) and are arranged as overlapping 1–2 seriate. They are phragmosporous, fusiform with conical ends, and initially pale yellowish-brown but becoming yellowish-brown as they mature. The ascospores are three septate and exhibit slight constriction at the septa. Their surfaces are rough walled. Asexual morph [36]: The *pycnidial conidiomata* are globose, black, erumpent, and possess a central ostiole. The *pycnidial wall* consists of 2–3 layers of brown textura angularis. *Conidiophores* are reduced to conidiogenous cells, which are hyaline, smooth, and doliiform. These cells exhibit prominent periclinal thickening or tightly aggregated percurrent proliferations. The *conidia* are solitary, brown, smooth, fusoid-ellipsoidal to subcylindrical, (1-)3 septate, and slightly constricted at the septa, with a subobtuse apex and a truncate base. They measure (8–)12–14(–16) × (2.5–)3(–3.5) µm.

Culture characteristics: After four weeks at 25 °C, colonies on potato dextrose agar (PDA) attained a diameter of up to 4 cm. These colonies exhibit an irregular, flattened to slightly raised morphology and display various color sectors ranging from white to creamy orange. The reverse side of the colonies appears creamy orange, with occasional dark patches that can be observed.

Known distribution: Crous et al. [36] identified this fungus in the Netherlands (specifically in Raalte), where it was found on *Poa* sp. (*Poaceae*) in a terrestrial habitat. In this study, we found it in China (specifically in Sichuan) on deceased bamboo culms (*Poaceae*). The habitat where it was discovered is terrestrial and typically covered with snow throughout the year.

Material examined: China, Sichuan, Dayi County, Xiling snow mountain, 30.684110 N, 103.164559 E, 3162 m, on dead bamboo, 06 January 2023, D.N. Wanasinghe, SCCSM23-012A-2 (HKAS 129196), living culture, KUNCC 23-13572. ibid. 30.684444 N, 103.164444 E, 2980 m, 6 January 2023, SCCSM23-012A-3 (HKAS 129195), living culture, KUNCC 23-13573.

Notes: The asexual form of *Phaeosphaeria poagena* was isolated from *Poa* sp. (*Poaceae*) and was introduced by Crous et al. [36]. *Phaeosphaeria poagena* is described as a new species because its small conidial dimensions do not match any of the asexual forms previously documented on *Poa* [32,33,37]. In our study, two strains from a sexually reproductive fungus formed a monophyletic clade with the type strain of *Phaeosphaeria poagena* (CBS 136771) with 99% MLB and 1.00 BYPP support values. A comparison of the ITS nucleotides between our new strains and the type strain of *Phaeosphaeria poagena* revealed only four base pair differences. Unfortunately, no protein-coding sequence data from the type strain are available. Instead of introducing a new species, we propose considering the host similarities (*Poaceae*) and ITS sequences to link these asexual and sexual forms as a single species. Therefore, we revised the species description to update the characteristics of its sexual form. This finding represents a new record of this fungus from bamboo and provides protein sequence data (*tef*1 and *rpb*2) for the species. Please refer to the notes under *Phaeosphaeria chengduensis* for further details.

**Figure 5:**
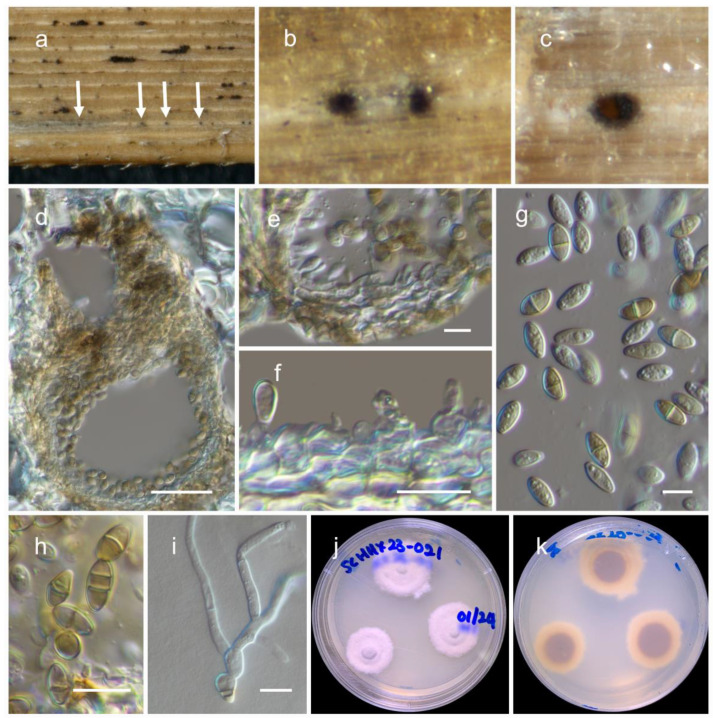
***Phaeosphaeria sichuanensis*** (HKAS 129194, holotype): (**a**,**b**) conidiomata on the dead *Pandanaceae* leaves (arrowed); (**c**) horizontal section of conidiomata; (**d**) vertical section through a conidioma; (**e**,**f**) pycnidial wall showing immature and mature conidia attached to conidiogenous cells; (**g**,**h**) conidia; (**i**) germinated conidium; (**j**,**k**) colonies on PDA after 6 weeks. Scale bars: (**d**) 50 µm; (**e**–**i**) 10 µm.

***Phaeosphaeria sichuanensis*** Wanas. & Maharachch. **sp. nov.** (Figure 5).

MycoBank: MB 849353.

*Etymology*: The specific epithet is derived from Sichuan, the location where this fungus was collected.

Holotype: HKAS 129194.

It is *saprobic* on dead *Pandanaceae* leaves. The sexual state was not observed. The asexual state: *conidiomata* are 140–170 μm high, 150–200 μm in diam., pycnidial, scattered and immersed, and appearing as slightly raised small black dots on the surface of the host. They are uniloculate and can be globose to subglobose or irregular in shape, with a color ranging from brown to dark brown. The *pycnidial wall* is 10–15 μm wide and consists of two layers of brown cells. The outer margin is composed of cells arranged in a textura angularis pattern, while the inner layer is made up of hyaline to pale brown cells arranged in a textura angularis pattern. The *conidiophores* are reduced to conidiogenous cells, which are holoblastic, phialidic, and ampulliform in shape and measure 3–6 × 3.5–5.5 μm (M = 4.6 × 4.4 μm, n = 20). They can be either hyaline or pale brown. The *conidia* measure 7–10 × 3.5–5 μm (M = 8.4 × 4 μm, n = 30) and are ovoid to ellipsoid in shape, with a conically rounded apex and base. Initially, the *conidia* are hyaline and aseptate with guttules, but they become pale brown to brown and 1-2 septate as they mature. Occasionally, they may contain guttules. The *conidia* are not constricted at the septa and have a smooth surface.

Culture characteristics: Colonies on PDA reach a diameter of 2 cm after four weeks at 25 °C. Initially, the colonies are white but become creamy white when mature. They are dense in texture, and slight radiation can be observed. The reverse side of the colonies appears creamy orange at the edges, gradually becoming orange-brown to brown at the center.

Known distribution: China (Sichuan) on dead *Pandanaceae* leaves in terrestrial habitats.

Material examined: China, Sichuan, Chengdu, Qingyang, Chengdu Huanhuaxi Park, 30.662011 N, 104.026047 E, 511 m, on dead twigs of an unknown deciduous host, 4 January 2023, D.N. Wanasinghe, SCHHX23-021-4 (HKAS 129194, holotype), ex-type culture, KUNCC 23-13569. ibid. 30.661886 N, 104.025530 E, 506 m, 4 January 2023, SCHHX23-021-3 (HKAS 129193), living culture, KUNCC 23-13568.

Notes: In our phylogenetic analysis, two strains (KUNCC 23-13568 and KUNCC 23-13569) from an asexual morphic fungus were found to be grouped with *Phaeosphaeria* species. Specifically, they formed the basal lineage of a monophyletic sister clade that included *Phaeosphaeria ampeli* (MFLUCC 19-0150 and MFLUCC 18-1641), *P. chengduensis* (KUNCC 23-13570 and KUNCC 23-13571), *P. chinensis* (KUMCC 19-0161 and MFLUCC 19-0217), *P. poagena* (KUNCC 23-13572 and KUNCC 23-13573), and *P. sinensis* (NCYUCC 19-0369). Morphologically, the conidia of the new fungus differ from other *Phaeosphaeria* species in terms of shape and the number of septa. Typically, *Phaeosphaeria* species produce fusiform conidia with multiple vertical septa [32,33]. However, this new fungus has ovoid to ellipsoid conidia with only 1–2 vertical septa. Nevertheless, the ampulliform, holoblastic, and conidiogenous cells of the new species bear resemblance to the asexual morphs in *Phaeosphaeria*.

***Septoriella*** Oudem., Ned. Kruidk. Arch. ser. 2, 5 (3): 504 (1889).

=*Allophaeosphaeria* Ariyaw., Camporesi & K.D. Hyde, Fungal Diversity 72: 137 (2015).

=*Dactylidina* Wanas., Camporesi & K.D. Hyde, Fungal Diversity 89: 107 (2018).

=*Hydeopsis* J.F. Zhang, J.K. Liu & Z.Y. Liu, Mycosphere 8: 211 (2019) syn. nov.

=*Naemostroma* Höhn., Berichte der Deutschen Botanischen Gesellschaft 37: 114 (1919).

=*Phaeopoacea* Thambug., Dissan. & K.D. Hyde, Mycosphere 8: 752 (2017) syn. nov.

=*Poaceicola* W.J. Li, Camporesi, Bhat & K.D. Hyde, Mycosphere 6 (6): 696 (2015).

=*Vagicola* Chethana & K.D. Hyde, Fungal Diversity 75: 113 (2015).

Notes: *Septoriella* was initially described by Oudemans [38], but it received limited attention, and only a few species were recognized. However, a recent study by Crous et al. [39] redefined the genus and established an epitype (CBS H-22281) and an ex-epitype strain (CBS 140065) for the type species, *Septoriella phragmitis*. The genus exhibits a wide distribution and encompasses a diverse array of species. *Septoriella* species are commonly associated with various host plants, including grasses, cereals, and other herbaceous plants [40]. Over the years, the taxonomy and classification of *Septoriella* have undergone significant revisions, with the discovery of new species and re-evaluation of existing ones using molecular and morphological data [40,41,42,43]. In the study of Marin-Felix [40], the ex-type strains of the sexual genera *Allophaeosphaeria*, *Poaceicola*, and *Vagicola* were found within the clade representing the genus *Septoriella*. Consequently, these genera were synonymized with *Septoriella* in their research. Subsequent to the research conducted by Crous et al. [39], later studies have contributed to the addition of 29 recognized species to the genus *Septoriella* [2]. However, it is crucial to note that this number is subject to change, as new species are continually being discovered and identified. Taxonomic studies have utilized various approaches, including molecular phylogenetics, morphology, and host specificity, to delineate and classify different species within *Septoriella*. In this study, we propose the synonymization of *Amarenographium ammophilicola*, *Amarenomyces dactylidis*, *Dactylidina shoemakeri*, *Hydeopsis verrucispora*, *Loratospora luzulae*, *Phaeopoacea asparagicola*, *P. festucae*, and *P. muriformis* under *Septoriella*.

***Septoriella ammophilicola*** (Dayar., E.B.G. Jones & K.D. Hyde) Wanas. & Maharachch., **comb. nov.**

MycoBank: MB 849361.

≡*Amarenographium ammophilicola* Dayar., E.B.G. Jones & K.D. Hyde, in Dayarathne et al., Mycosphere 11(1): 59 (2020).

Holotype: UK, Wales, Carmarthenshire, Cefn Sidan, on a leaf of Marram grass (*Ammophila arenaria*) in a sand dune, 15 October 2017, E.B. Gareth Jones, GJ448 (MFLU 17-2571, holotype).

Descriptions and illustrations. See Dayarathne et al. [44].

Notes: Dayarathne et al. [44] introduced *Amarenographium ammophilicola* based on its morphological characteristics and phylogenetic analysis. In our phylogenetic analysis, the type strain of *A*. *ammophilicola* clustered with other *Septoriella* species within the *Phaeosphaeriaceae* (Figure 1). Therefore, we propose the transfer of *Amarenographium ammophilicola* to the genus *Septoriella*, and subsequently it should be recognized as *Septoriella ammophilicola*, comb. nov.

***Septoriella asparagicola*** (Phukhams., Akulov & K.D. Hyde) Wanas. & Maharachch., **comb. nov.**

MycoBank: MB 849362.

≡*Phaeopoacea asparagicola* Phukhams., Akulov & K.D. Hyde, in Hyde et al., Fungal Diversity 96: 57 (2019).

Holotype: Ukraine, Odessa Region, Lyman District, Tiligulskyt Regional Landscape Park, on the overwintered stems of *Asparagus* sp., 1 May 2014, A. Akulov, EX CWU (MYC) AS 5825 (MFLU 18-1380, holotype), ex-type MFLUCC 16-0379.

Descriptions and illustrations: See Hyde et al. [19].

Notes: Thambugala et al. [42] established the genus *Phaeopoacea* to accommodate *P. festucae* and *P. phragmiticola* (≡*Phaeosphaeria phragmiticola*). The type species, *P. festucae*, was previously known only from its asexual morph, which forms pycnidial conidiomata that are globose to subglobose or linear in rows on the host, producing brown to dark brown, oblong conidia [42]. Meanwhile, *Phaeosphaeria phragmiticola* was previously considered a synonym of *Septoriella leuchtmannii* by Crous et al. [39]. However, based on multi-gene phylogenetic analyses conducted by Li et al. [41] and Thambugala et al. [42], *Phaeosphaeria phragmiticola* was placed within *Phaeopoacea*. Hyde et al. [19,45] later included two additional species, *P. asparagicola* and *P. muriformis*, in this genus. The phylogenetic analysis by various authors revealed that *Phaeopoacea* does not form a clearly defined clade and consistently clusters with the genera *Allophaeosphaeria*, *Amarenographium*, *Amarenomyces*, *Dactylidina*, *Poaceicola*, *Septoriella*, and *Vagicola* [35]. In our phylogenetic analysis, the three type strains of *Phaeopoacea*, *P. asparagicola* (MFLUCC 16-0379), *P. festucae* (MFLUCC 17-0056), and *P. muriformis* (MFLUCC 17-0372) were found to be distantly related to each other within *Septoriella*. To provide better clarity and avoid unresolved taxonomic circumscriptions, we propose the synonymization of these three species under *Septoriella*. As a result, we established a new combination by classifying *Phaeopoacea asparagicola* within *Septoriella*.

***Septoriella festucae*** (Dissan. & K.D. Hyde) Wanas. & Maharachch., **comb. nov.**

MycoBank: MB 849363.

≡*Phaeopoacea festucae* Dissan. & K.D. Hyde, in Thambugala et al., Mycosphere 8(4): 752 (2017).

Holotype: Italy, Province of Forlì-Cesena, near Santa Sofia, on a dead aerial stem of *Festuca pratensis*, 16 July 2013, Erio Camporesi IT 1384 (MFLU 17– 0121), ex-type MFLUCC 17–0056.

Descriptions and illustrations: See Thambugala et al. [42].

Notes: Our study findings have led us to propose the reclassification of *Phaeopoacea* into the genus *Septoriella*, resulting in the establishment of *Septoriella festucae*, comb. nov. For more information, please refer to the note provided under *Septoriella asparagicola*.

***Septoriella luzulae*** (Jayasiri, Camporesi & K.D. Hyde) Wanas. & Maharachch., **comb. nov.**

MycoBank: MB 849364.

≡*Loratospora luzulae* Jayasiri, Camporesi & K.D. Hyde, in Ariyawansa et al., 75: 108 (2015).

Holotype: Italy, Province of Forlì-Cesena, Campigna-Santa Sofia, on dead stems of *Luzula nivea*, 8 June 2014, E. Camporesi, IT 1918 (MFLU 15-1394, holotype), ex-type MFLUCC 14-0826.

Descriptions and illustrations: See Ariyawansa et al. [46].

Notes: The monotypic genus *Loratospora* was established by Kohlmeyer and Volkmann-Kohlmeyer [47] to accommodate *L*. *aestuarii*. Later, Ariyawansa et al. [46] introduced *Loratospora luzulae* as the second species in this genus. Although their phylogenetic analysis showed that these two species were closely related, they were not monophyletic (page 98, Figure 40). In this study, the type strain of *Loratospora luzulae* (MFLUCC 14-0826) grouped with *Septoriella* species, particularly showing a sister relationship to *Septoriella callistemonis* (CBS 146822) and *S. camporesii* (KUMCC 16-0113). Therefore, we have assigned *Loratospora luzulae* to the *Septoriella* genus as a new combination (*S. luzulae*).

***Septoriellaneomuriformis*** Wanas. & Maharachch., **nom. nov.**

MycoBank: MB 849365.

≡*Phaeopoacea muriformis* Karun. & K.D. Hyde, in Hyde et al., Fungal Diversity 87: 83 (2017).

Holotype: China, Yunnan Province, Kunming Institute of Botany, Botanical Garden, on stems of unidentified grass, 28 November 2016, K.V.A. Karunarathna, AKKIB 49 (MFLU 17-0372, holotype; HKAS 97365, isotype), ex-type living cultures MFLUCC 17-1382, KUMCC 16-0234.

Descriptions and illustrations: See Hyde et al. [45].

Notes: Our study findings have led us to propose the reclassification of *Phaeopoacea* into the genus *Septoriella*, resulting in the establishment of *Septoriella muriformiae*, nom. nov. Since the name ‘*Septoriella muriformis*’ is already in use within the *Septoriella* genus, we have employed a nomenclature novelty (*Septoriella neomuriforme*) for this synonymization. For more information, please refer to the note provided under *Septoriella asparagicola*.

**Figure 6:**
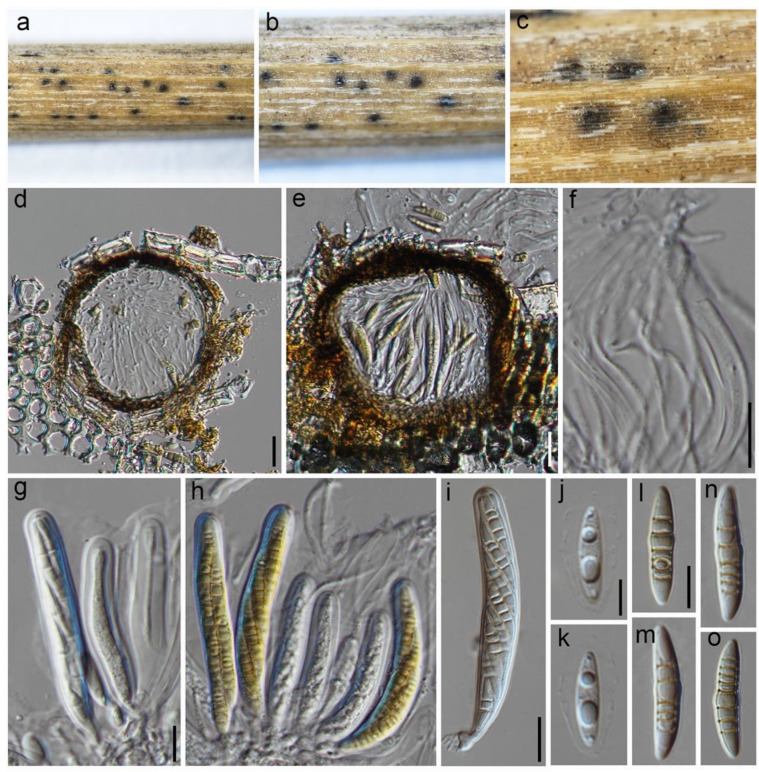
***Septoriella paradactylidis*** (HKAS 129216): (**a**–**c**) ascomata on the host; (**d**,**e**) cross section of ascomata; (**f**) pseudoparaphyses; (**g**–**i**) asci; (**j**–**o**) ascospores. Scale bars: (**d**,**f**,**g**,**i**) 20 µm; (**j**,**l**) 10 µm, scale bar of (**d**) applies to (**e**), scale bar of (**g**) applies to (**g**–**i**), scale bar of (**j**) applies to (**k**), and scale bar of (**l**) applies to (**l**–**o**).

***Septoriella paradactylidis*** Wanas. & Maharachch., **nom. nov.** (Figure 6).

MycoBank: MB 849366.

≡*Amarenomyces dactylidis* Mapook, Camporesi & K.D. Hyde, in Hyde et al., Fungal Diversity 87: 78 (2017).

It is *saprobic* on decaying clumps of *Lolium perenne*. The sexual morph: *Ascomata* are 120–190 μm high and 130–200 μm diam., are semi-immersed to erumpent, solitary, scattered, uniloculate, and have a globose to subglobose shape. They are dark brown in color, with a central ostiole and a minute papilla. The *peridium* is 13–25 μm wide and consists of 3–6 layers of brown to dark brown cells with a textura angularis structure. The *hamathecium* is made up of filamentous, cylindrical to filiform, septate pseudoparaphyses that are 2–4 μm wide and are embedded in a gelatinous matrix. The *asci* measure (70–)75–90(–110) × (9–)10–12(–15) μm (x = 83 × 9.5 μm, n = 10), with a developed ocular chamber. They are eight-spored, bitunicate, fissitunicate, cylindrical, and short pedicellate. The *ascospores* measure 20–27 × 5–6 μm (x = 24.5 × 5.5 μm, n = 40) and are broadly fusiform to inequilateral in shape. They are hyaline to pale yellowish, 5–8 septate, with the widest part at the middle cell. The *ascospores* are asymmetrical and have a smooth wall, surrounded by a hyaline gelatinous sheath. The asexual state was not observed.

Culture characteristics: The ascospores showed germination on PDA within 24 h. After a two-week incubation period at 25 °C, the colonies on PDA medium grew to a diameter of 4 cm. These colonies had an undulate margin and initially appeared whitish grey, transitioning to a grey shade with an irregularly raised texture at the center. Towards the periphery, the color became greenish grey. When observed from the reverse side, the colonies displayed a blackish brown color at the center and a creamy grey coloration towards the periphery.

Known distribution: China, Italy, on *Dactylis glomerata*, *Lolium perenne*, and unknown fern.

Material examined: China, Sichuan, Chengdu, Chenghua District, The Chengdu Research Base of Giant Panda Breeding, on dead culms of *Lolium perenne* L., 13 August 2021, Q Wang, W90-3 (HKAS 129216), culture, KUNCC 23-14219.

Notes: Our phylogenetic analyses have revealed that the new strain KUNCC 23-14219 is closely related to the other isolates of *Septoriella paradactylidis* (=*Amarenomyces dactylidis*), including the type strain (MFLU 17-0498). Further investigations comparing our isolate to the type species have revealed a similar size range of the ascomata, asci, and ascospores, as well as the ascospore septation [45]. Therefore, we are now reporting the presence of *S. neodactylidis* in *Lolium perenne*, which represents the first record of this fungus in that host and provides protein sequence data (*tef*1 and *rpb*2) for this species. Since the name ‘*Septoriella dactylidis*’ is already in use within the *Septoriella* genus, we have utilized a nomenclature novelty (*Septoriella paradactylidis*) for this synonymization.

***Septoriella shoemakeri*** Wanas. & Maharachch., **sp. nov.**

MycoBank: MB 849358.

≡*Dactylidina shoemakeri* Wanas., Camporesi, E.B.G. Jones & K.D. Hyde, in Wanasinghe et al., Fungal Diversity 89: 109 (2018).

Holotype: Italy, Trento, Marilleva 1400, on dead aerial stems of *Poa* sp., 2 August 2013, Erio Camporesi IT 1932 (MFLU 16-0202, holotype); ex-type MFLUCC 14-0963.

Descriptions and illustrations: See Wanasinghe et al. [48].

Notes: In Wanasinghe et al. [48], they described *Dactylidina shoemakeri* based on its morphological traits and a conducted phylogenetic analysis. However, it should be noted that this species has not been validly published (Nom. inval., Art. 35.1 (Shenzhen)). In our phylogenetic analysis, we found that the strain MFLUCC 14-0963, which was previously invalidly published as *Dactylidina shoemakeri*, grouped with *Septoriella* species and showed a close affiliation to *S. neodactylidis* (MFLUCC 14-0966). Therefore, we propose reassigning *Dactylidina shoemakeri* to the genus *Septoriella*, and going forward, it should be referred to as *Septoriella shoemakeri*.

***Septoriella verrucispora*** (J.F. Zhang, J.K. Liu & Z.Y. Liu) Wanas. & Maharachch., **comb. nov.**

MycoBank: MB 849368.

≡*Hydeopsis verrucispora* J.F. Zhang, J.K. Liu & Z.Y. Liu, in Zhang et al., Mycosphere 8(1): 211 (2019).

Holotype: China, Guizhou Province, Guiyang City, Huaxi District, dead culms of herbaceous plant, 3 April 2016, J.F. Zhang, SD-2016-5 (MFLU 18-2269; holotype); ex-type MFLUCC 19-0163, GZCC 19-0001.

Descriptions and illustrations: See Zhang et al. [49].

Notes: The monotypic genus *Hydeopsis*, typified by *H*. *verrucispora*, was introduced by Zhang et al. [49]. It exhibited a close phylogenetic relationship with *Dactylidina* and *Phaeopoacea* within the family *Phaeosphaeriaceae*. The authors differentiated *Hydeopsis* from these genera based on differences in pigmentation and the number of septa in ascospores. Our combined sequence phylogenetic analysis reveals that *Hydeopsis verrucispora* is phylogenetically positioned within the genus *Septoriella*. Additionally, pigmentation and the number of septa appear to be highly informative at the species level, although these characteristics alone cannot reliably distinguish between genera. Species of *Septoriella* also share more similar characteristics. Therefore, *Hydeopsis* is determined to be congeneric with *Septoriella*, and as a result, we propose assigning it to the genus *Septoriella* as a new combination.

## 4. Discussion

The delimitation of species in *Phaeosphaeriaceae* is typically based on a combination of morphological and molecular characteristics. Some of the major morphological features used to delimitate species in this family include fruiting body type, peridium, spores, asci, conidiogenous cells, and colony morphology [4]. The shape, size, and color of ascospores and conidia can vary between different species of *Phaeosphaeriaceae*. However, the usefulness of these morphological features for species delimitation can vary depending on the species and the taxonomic group being studied. There are several challenges in using morphological features for species delimitation in *Ascomycota*, including in *Phaeosphaeriaceae*. Morphological variation within species of *Phaeosphaeriaceae* can pose challenges in distinguishing closely related species. For instance, in the case of *Septoriella* species, there are variations in ascospore features [40]. These include the arrangement of 1–3 seriate in an ascus, colors ranging from hyaline to yellowish-brown, pale brown, brown, golden brown, or reddish-brown, and shapes that can be narrowly or broadly fusiform, oblong, or narrowly oblong. The surface can be smooth-walled or echinulate, with transverse or longitudinal septa and sometimes enlarged medium cells. The ends of the ascospores can be conical, obtuse, or rounded, and they may or may not have sheaths. Furthermore, even the conidia morphology of the asexual morphs within these species can vary [39], with shapes ranging from cylindrical to subcylindrical, fusiform, or subfusiform. The apex can be obtuse or subobtuse, the base truncate, and the conidia can be straight or curved. They are euseptate, pale brown to brown, and can have thin-walled, smooth surfaces or minutely verruculose surfaces. Additionally, they may bear mucoid appendages at both ends.

Cryptic or polyphyletic species within *Phaeosphaeriaceae* are morphologically similar but genetically distinct, making it challenging to differentiate them based solely on morphology. This can be observed in sexual morphs resembling *Phaeosphaeria* or *Ophiobolus*, as well as asexual morphs with a camarosporium-like resemblance (i.e., *Melnikia*, *Camarosporioides*, and *Dlhawksworthia*). Convergence, where different species develop similar traits due to similar ecological pressures, can contribute to this morphological similarity. Moreover, variations in methods used to observe and measure morphological features can lead to inconsistencies in species delineation. Careful examination of old literature is crucial to avoid mistakenly introducing existing species as new ones. Additionally, limited information on the morphological features of certain *Phaeosphaeriaceae* species in old-type materials makes accurate delimitation based on morphology alone difficult. To overcome these challenges, a combination of morphological and molecular approaches is essential for species delineation in *Phaeosphaeriaceae*. Molecular data can provide valuable insights into genetic variation and evolutionary relationships, aiding in the resolution of taxonomic uncertainties and differentiation between closely related species with similar morphology.

The taxonomy of the *Phaeosphaeria* genus is continuously evolving, with ongoing research and the utilization of molecular techniques aiding in the identification and classification of new species. Molecular phylogenetic analyses, in conjunction with morphological studies, have contributed to the clarification of species boundaries and relationships within the genus. The diversity of *Phaeosphaeria* is evident through the wide range of hosts, substrates, and ecological roles displayed by its various species. Recent phylogenetic analyses have revealed that *Phaeosphaeria* is polyphyletic, leading to the reclassification of many *Phaeosphaeria* sensu lato species into different genera within *Phaeosphaeriaceae* [37,40,43,45,46,50,51].

Based on our multi-gene phylogenetic analysis, strains of *Phaeosphaeria* were found to be distributed across several subclades within *Phaeosphaeriaceae*. Among all the *Phaeosphaeria* strains examined, only *P. orae-maris* (CBS 255.64: MH858433 and MH870063) displayed a distant relationship from the family *Phaeosphaeriaceae* in our primary analyses. Instead, it exhibited a close phylogenetic relationship to *Lentitheciaceae*. For our phylogenetic analysis, we included all the *Phaeosphaeria* strains that formed a monophyletic group with the type strain (*Phaeosphaeria oryzae*). Additionally, we incorporated other *Phaeosphaeria* strains that were closely associated with *Paraloratospora* and *Septoriella*. However, the remaining strains that showed close affiliations with other genera, namely *Phaeosphaeria anchiala* (5552A and 5547D), *P. avenaria* (AFTOL-ID 280), *P. breonadiae* (CPC 25944), *P. eustoma* (AFTOL-ID 1570), *P. fructigena* (FMR 17808), *P. fusispora* (LC6215 and LC5367), *P. halima* (RKDO787 and RKDO844), *P. juncicola* (CBS 110108), *P. nardi* (CBS 304.71), *P. nigrans* (CBS 307.79 and CBS 576.86), *P. occulta* (CBS 582.86), *P. olivacea* (AFTOL-ID 2206 and JK 5540Q), *P. orae-maris* (AFTOL-ID 1441), *P. spartinae* (CBS 254.64, RKDO785, RKDO806, and RKDO808), *P. spartinicola* (CBS 176.91, CBS 118215, and JK 5177A), and *P. typharum* (CBS 296.54), were excluded from the final analysis. These decisions were made to ensure the accuracy and validity of our phylogenetic analysis, focusing specifically on the relationships within the genera *Paraloratospora*, *Phaeosphaeria*, and *Septoriella*.

Strains belong to eighteen existing *Phaeosphaeria* species, namely *P. acaciae* (KUMCC 20-0214 and MFLU 17-0496), *P. ampeli* (MFLUCC 18-1641 and MFLUCC 19-0150), *P. calamicola* (MFLUCC 14-1168), *P. caricis-sectae* (CBS 146823), *P. chiangraina* (MFLUCC 13-0231), *P. chinensis* (KUMCC 19-0161 and MFLUCC 19-0217), *P. cycadis* (KUMCC 18-0161 and KUMCC 18-0162), *P. lunariae* (CPC 26679), *P. musae* (CBS 120026, MFLUCC 11-0133, MFLUCC 11-0151, and MFLUCC 17-2648), *P. nodulispora* (URM 7220), *P. oryzae* (CBS 110110 and MFLUCC 11-0170), *P. papayae* (CBS 135416 S528), *P. penniseti* (FU31020), *P. phoenicicola* (CPC 28711), *P. poagena* (CBS 136771), *P. podocarpi* (CBS 138903), *P. sinensis* (KUMCC 17-0195, MFLUCC 18-1552, and NCYUCC 19-0369), and *P. thysanolaenicola* (MFLUCC 10-0563), which are grouped in *Phaeosphaeria* sensu stricto (Figure 1). Additionally, two of our new strains (KUNCC 23-13572 and KUNCC 23-13573) were grouped with the type strain of *Phaeosphaeria poagena* (CBS 136771), and four new strains formed two distinct monophyletic lineages that we have introduced as two new species, namely *Phaeosphaeria chengduensis* and *P. sichuanensis*.

Based on our multi-gene phylogenetic analysis, we observed that *Paraloratospora* is closely related to *Loratospora aestuarii* (JK 5535B), *Sulcispora pleurospora* (CBS 460.84), *S. supratumida* (MFLUCC 14-0995), and *Wingfieldomyces cyperi* (CBS 141450), forming a sister relationship. However, it is important to note that these species were represented by single strains. Therefore, further evaluation of their inter-generic relationships should be conducted with additional species/collections and through DNA-based sequence data analyses. Currently, only three species are accepted in *Paraloratospora*, viz., *P. camporesii*, *P. gahniae*, and *P. marina* [52]. In our analysis, two type strains of *Phaeosphaeria breonadiae* (CPC 25944) and *P. fructigena* (FMR 17808) were grouped among these three *Paraloratospora* species. Additionally, two of our new strains (KUNCC 23-14218 and HKAS 129218) also clustered with *Paraloratospora* species. Consequently, we synonymized *Phaeosphaeria breonadiae* and *P. fructigena* under *Paraloratospora*, and introduced our new strains as belonging to a new species. Furthermore, strains provisionally named as *Phaeosphaeria avenaria* f. sp. *tritici* (CBS 289.52), *P. caricicola* (CBS 603.86), *P. eustoma* (CBS 307.71 and CBS 724.92), *P. glyceriae-plicatae* (CBS 101261), *P. juncophila* (CBS 575.86), *P. norfolcia* (CBS 593.86), and *P. parvula* (CBS 260.49 and CBS 605.86) were also grouped within the genus *Paraloratospora*. However, since these strains are not related to their type materials, we did not transfer these species to *Paraloratospora* in our analysis. Further studies are needed to resolve the phylogenetic classification of the aforementioned species.

In our analysis, we primarily utilized single-gene phylogenetic analyses but obtained a more robust topology through the combined gene analysis (SSU + LSU + ITS + *tef*1 + *rpb*2). For molecular comparisons, ITS sequences were available for 97.7% of the species in the family, while LSU sequences were available for 98.8%. However, the availability of sequence data for protein-coding genes, such as *tef*1 and *rpb*2, was limited in previous studies, with *tef*1 available for 54.6% of the species and *rpb*2 available for 32.7%. By incorporating more informative gene data and conducting extensive sampling worldwide, it is anticipated that most of the monotypic species will be transferred to a few genera within the family. One example of this is the synonymization of *Hydeopsis* under *Septoriella* in this study. Based on morphological, phylogenetic, and ecological evidence, we have introduced four new species, seven new combinations, and two new names in the genera *Paraloratospora*, *Phaeosphaeria*, and *Septoriella*. Despite the advancements in our understanding of these genera, several challenges still persist. Differentiating closely related species remains a significant obstacle, necessitating further investigation and the integration of multiple data sources. Moreover, the limited knowledge regarding the life cycles, host range, and geographic distribution of many of these species hinders our comprehension of their ecological roles. To overcome these challenges, future research should prioritize the utilization of advanced molecular techniques such as genomics, transcriptomics, and metagenomics. These approaches can provide insights into the genetic diversity, evolutionary history, and ecological functions of *Paraloratospora*, *Phaeosphaeria*, and *Septoriella*. By employing these cutting-edge methods, we can unravel the intricate complexities within these genera and gain a more comprehensive understanding of their significance in the ecosystem.

## Figures and Tables

**Table 1:** Taxa used in the phylogenetic analyses and their corresponding GenBank numbers. Isolates/sequences in bold were isolated/sequenced in the present study. The superscripted “T” in the strain column denotes ex-type, ex-isotype, ex-paratype, or ex-epitype strains.

Species	Strain	GenBank Accession Numbers				
		ITS	LSU	SSU	*tef*1	*rpb*2
*Acericola italica*	MFLUCC 13-0609 ^T^	NR_156344	NG_057143	NG_063642	-	-
*Alloconiothyrium camelliae*	NTUCC 17-032-1	MT112294	MT071221	MT071270	MT232967	-
*Alloconiothyrium camelliae*	NTUCC 17-032-2	MT112295	MT071222	MT071271	MT232965	-
*Alloneottiosporina thailandica*	MFLUCC 15-0576 ^T^	NR_169715	NG_073831	NG_070332	MT454002	MT432196
*Amarenomyces ammophilae*	CBS 114595	KF766146	GU301859	GU296185	GU349035	GU371724
*Arezzomyces cytisi*	MFLUCC 15-0649 ^T^	NR_153533	NG_057083	NG_063584	-	-
*Banksiophoma australiensis*	CBS 142163 ^T^	KY979739	KY979794	-	-	-
*Bhagirathimyces himalayensis*	NFCCI 4580 ^T^	NR_168816	NG_068644	NG_068414	MT091965	-
*Bhatiellae rosae*	MFLUCC 17-0664 ^T^	NR_157505	NG_058718	NG_061286	-	-
*Brunneomurispora lonicerae*	KUMCC 18-0157 ^T^	NR_164299	NG_070462	NG_067697	MK359064	MK359079
*Brunneomurispora lonicerae*	KUMCC 18-0158	MK356373	MK356347	MK356361	MK359065	-
*Camarosporioides phragmitis*	MFLUCC 13-0365 ^T^	NR_153925	NG_058954	KX572350	KX572354	-
*Chaetosphaeronema clematidicola*	MFLUCC 17-2151 ^T^	MT310619	MT214574	NG_070660	MT394633	MT394694
*Chaetosphaeronema clematidis*	MFLUCC 17-2147	MT310620	MT214575	MT226688	MT394634	MT394695
*Chaetosphaeronema hispidulum*	CBS 216.75	KF251148	KF251652	EU754045	-	GU371777
*Dematiopleospora cirsii*	MFLUCC 13-0615 ^T^	KX274243	KX274250	-	KX284708	-
*Dematiopleospora mariae*	MFLU 16-0121	MT310621	MT214576	MT226689	MT394635	-
*Dematiopleospora mariae*	MFLUCC 13-0612 ^T^	KX274244	KJ749653	KJ749652	KJ749655	-
*Dematiopleospora salsolae*	MFLUCC 17-0828 ^T^	NR_157514	NG_059184	NG_063679	MG829201	MG829254
*Didymocyrtis consimilis*	CBS 129141	KT383812	KT383796	-	-	-
*Didymocyrtis ramalinae*	Ertz 16399	KT383838	KT383802	-	-	-
*Diederichomyces caloplacae*	CBS 129140	JQ238635	JQ238637	-	-	-
*Diederichomyces caloplacae*	CBS 129338	JQ238641	JQ238643	-	-	-
*Diederichomyces ficuzzae*	CBS 128019	KP170647	JQ238616	-	-	-
*Dlhawksworthia alliariae*	MFLUCC 13-0070 ^T^	NR_164244	NG_069375	KX494878	-	KX507261
*Dlhawksworthia clematidicola*	MFLUCC 14-0910 ^T^	NR_164267	NG_069556	NG_063681	MG829202	-
*Dlhawksworthia lonicera*	MFLUCC 14-0955 ^T^	NR_164268	NG_069557	NG_063682	MG829203	-
*Edenia gomezpompae*	CBS 124106 ^T^	NR_156217	NG_059202	-	-	-
*Edenia gomezpompae*	LVPEI 3225	KU578033	-	-	-	-
*Elongaticollum hedychii*	MFLUCC 18-1638	MT321796	MT321810	MT321803	MT328753	-
*Elongaticollum hedychii*	MFLUCC 17-2653 ^T^	MT321797	MT321811	MT321804	MT328754	-
*Elongaticollum hedychii*	NCYUCC 19-0286	MT321798	MT321812	MT321805	MT328755	-
*Embarria clematidis*	MFLUCC 14-0652 ^T^	KT306949	KT306953	KT306956	-	-
*Embarria clematidis*	MFLUCC 14-0976	MG828871	MG828987	MG829099	MG829194	-
*Equiseticola fusispora*	MFLUCC 14-0522 ^T^	NR_154010	NG_059249	NG_061238	MG520895	-
*Galiicola baoshanensis*	HKAS 102234 ^T^	NR_164300	NG_068613	NG_067698	MK359066	-
*Galiicola dactylidicola*	MFLUCC 13-0738 ^T^	NR_154501	NG_067531	NG_063564	-	-
*Galiicola pseudophaeosphaeria*	MFLU 14-0524 ^T^	KT326692	KT326693	-	MG520896	-
*Hydeomyces desertipleosporoides*	SQUCC 15259 ^T^	NR_164295	NG_068287	NG_067692	MK290848	-
*Hydeomyces desertipleosporoides*	SQUCC 15260	MK290842	MK290840	MK290844	MK290849	-
*Hydeomyces hydei*	SQUCC 15290 ^T^	NR_173311	MW077157	NG_074955	MW075774	MW276078
*Italica achilleae*	MFLUCC 14-0959 ^T^	NR_157518	NG_059862	NG_063683	MG829204	MG829255
*Italica heraclei*	MFLU 18-1906 ^T^	MT881676	MT881653	MT881671	MT901290	-
*Jeremyomyces labinae*	CBS 144617 ^T^	NR_163362	NG_070463	-	-	MK442665
*Juncaceicola alpina*	CBS 456.84 ^T^	NR_145172	NG_069801	NG_063078	-	-
*Juncaceicola luzulae*	MFLUCC 13-0780 ^T^	KX449529	KX449530	KX449531	MG520898	-
*Juncaceicola padellana*	CBS 606.86 ^T^	NR_145138	NG_069810	-	-	-
*Kwanghwaensis miscanthi*	FU31017 ^T^	MK503817	MK503823	MK503829	MT009126	-
*Leptospora clematidis*	MFLUCC 17-2148 ^T^	NR_170811	MT214577	MT226690	MT394636	MT394696
*Leptospora galii*	KUMCC 15-0521 ^T^	NR_168158	NG_068778	NG_068364	MG520899	-
*Leptospora hydei*	GZCC 19-0004 ^T^	MK522507	MK522497	MK522503	MK523387	-
*Longispora clematidis*	MFLU 15–1277 ^T^	MT310625	MT214580	MT226693	MT394639	-
*Loratospora aestuarii*	JK 5535B	MH863024	GU301838	GU296168	-	GU371760
*Mauginiella scaettae*	CBS 239.58	MH857770	MH869303	-	-	-
*Mauginiella scaettae*	CBS 158.26	MH854874	MH866370	-	-	-
*Megacoelomyces sanchezii*	CMHUB 24030	MN429018	MN429020	MN429016	-	MN546863
*Megacoelomyces sanchezii*	CMHUB 24055	MN429019	MN429021	MN429017	-	-
*Melnikia anthoxanthi*	MFLUCC 14-1011 ^T^	-	KU848204	KU848205	-	-
*Murichromolaenicola chiangraiensis*	MFLUCC 17-1488 ^T^	MN994582	MN994559	MN994605	MN998163	-
*Murichromolaenicola chromolaenae*	MFLUCC 17-1489 ^T^	MN994583	MN994560	MN994606	MN998164	-
*Muriphaeosphaeria galatellae*	MFLUCC 14-0614 ^T^	NR_154189	NG_059600	NG_061216	MG520900	-
*Muriphaeosphaeria galatellae*	MFLUCC 15-0769	-	KT438330	KT438332	-	-
*Neoophiobolus chromolaenae*	MFLUCC 17-1467 ^T^	NR_168851	NG_068693	NG_070144	MN998166	-
*Neoophiobolus chromolaenae*	MFLUCC 17-1449	MN994584	MN994561	MN994607	MN998165	-
*Neosetophoma camporesii*	MFLUCC 15-0682 ^T^	KU302779	KU302778	NG_070320	MN654114	-
*Neosetophoma garethjonesii*	MFLUCC 14-0528 ^T^	KY496758	KY496738	KY501126	KY514402	-
*Neosetophoma rosae*	MFLUCC 17-0844	MG828926	MG829035	MG829141	MG829219	-
*Neosetophoma shoemakeri*	MFLUCC 17-2510 ^T^	NR_161044	NG_069548	NG_065723	MG739515	-
*Neosphaerellopsis thailandica*	CPC 21659 ^T^	NR_137954	NG_067289	-	-	-
*Neostagonospora arrhenatheri*	MFLUCC 15-0464 ^T^	KX926417	KX910091	KX950402	MG520901	-
*Neostagonospora caricis*	CBS 135092 ^T^	NR_156264	NG_070457	-	-	KF252171
*Neostagonospora elegiae*	CBS 135101 ^T^	NR_156265	NG_070369	-	-	KF252172
*Neostagonospora phragmitis*	MFLUCC 16-0493 ^T^	KX926416	KX910090	KX950401	MG520902	-
*Neostagonospora sorghi*	CBS 145366 ^T^	NR_164452	NG_070466	-	-	MK540087
*Neostagonospora spinificis*	R. Kirschner 3867	KP676045	KP676046	-	-	-
*Neostagonosporella bambusicola*	KUMCC 20-0031 ^T^	OP454331	OP454335	OP454058	-	-
*Neostagonosporella sichuanensis*	MFLUCC 18-1228 ^T^	MH368073	MH368079	MH368088	MK313851	-
*Neostagonosporella sichuanensis*	MFLU 18-1223	MH394690	MH394687	MK296469	MK313854	-
*Neosulcatispora agaves*	CPC 26407 ^T^	NR_154233	NG_070394	-	-	-
*Nodulosphaeria aconiti*	MFLUCC 13-0728 ^T^	NR_154236	KU708844	KU708840	KU708852	KU708856
*Nodulosphaeria digitalis*	MFLUCC 17-2418	MG891749	MG891750	MH791042	MH791041	-
*Nodulosphaeria guttulatum*	MFLUCC 15-0069	KY496746	KY496726	KY501115	KY514394	KY514405
*Nodulosphaeria scabiosae*	MFLUCC 14-1111 ^T^	NR_154237	KU708846	NG_063602	KU708854	KU708857
*Ophiobolopsis italica*	MFLUCC 17-1791 ^T^	NR_156678	NG_059856	NG_061281	MG520903	-
*Ophiobolus disseminans*	MFLUCC 17-1787	MG520941	MG520961	MG520980	MG520906	-
*Ophiobolus hydei*	TASM 6143	MK981301	MK981305	MK981303	MK993651	-
*Ophiobolus hydei*	TASM 6144	MK981300	MK981304	MK981302	MK993650	-
*Ophiosimulans tanaceti*	MFLUCC 14-0525	KU738890	KU738891	KU738892	MG520910	-
*Ophiosphaerella taiwanensis*	NCYUCC 19-0152 ^T^	NR_171874	MT321815	MT321808	MT328758	-
*Ophiosphaerella taiwanica*	NTUCC 17-024 ^T^	NR_171846	MN082419	-	MN199124	-
*Ophiosphaerella taiwanica*	NTUCC 17-025	MN082418	MN082420	-	MN199125	-
*Paraleptospora chromolaenae*	MFLUCC 17-1481 ^T^	NR_168852	NG_068694	NG_070145	MN998167	-
*Paraleptospora chromolaenicola*	MFLUCC 17-2670 ^T^	NR_168853	NG_068695	NG_070146	MN998168	-
*Paraloratospora breonadiae*	CPC 25944 ^T^	NR_155675	NG_068582	-	-	-
*Paraloratospora camporesii*	MFLU 18-0915 ^T^	NR_170014	NG_073784	MN756635	MN756633	-
*Paraloratospora fructigena*	FMR 17808 ^T^	OU612363	OU612362	-	OU600609	OU600607
*Paraloratospora gahniae*	CPC 32454 ^T^	NR_156675	NG_059852	-	-	MG386148
*Paraloratospora marina*	MFLUCC 19 0691 ^T^	OQ130046	OQ130110	OQ130107	OQ357219	OQ162221
***Paraloratospora sichuanensis***	**KUNCC 23-14218** **^T^**	**OR206396**	**OR206415**	**OR206405**	**OR195712**	**OR195721**
***Paraloratospora sichuanensis***	**HKAS 129218**	**OR206397**	**OR206416**	**OR206406**	**OR195713**	**OR195722**
*Paraophiobolus arundinis*	MFLUCC 17-1789 ^T^	NR_156680	NG_069539	NG_061282	MG520912	-
*Paraophiobolus torilicola*	MFLU 20-0392	MT370411	MT370428	MT370372	-	-
*Paraphoma chrysanthemicola*	CBS 522.66	KF251166	KF251670	GQ387521	-	KF252174
*Paraphoma ledniceana*	CBS 146533 ^T^	NR_172538	MT371396	-	-	-
*Paraphoma radicina*	CBS 111.79 ^T^	NR_156556	NG_070446	EU754092	-	KF252180
*Paraphoma rhaphiolepidis*	CBS 142524 ^T^	KY979758	NG_070415	-	-	KY979851
*Paraphoma vinacea*	UMPV001	KU176884	KU176888	-	-	-
*Parastagonospora fusiformis*	MFLUCC 13-0215 ^T^	NR_165848	NG_068235	NG_068367	-	KX863711
*Parastagonospora nodorum*	CBS 259.49 ^T^	KF251185	KF251688	-	GU456285	KF252192
*Parastagonospora phragmitis*	CPC 32075 ^T^	NR_164454	NG_066451	-	-	MK540089
*Parastagonospora poaceicola*	MFLUCC 15-0471 ^T^	-	NG_068537	NG_068368	-	KX880499
*Parastagonosporella fallopiae*	CBS 135981 ^T^	NR_160062	NG_067337	-	-	MH460547
*Parastagonosporella fallopiae*	CCTU 1151-1	MH460544	MH460546	-	-	-
*Phaeosphaeria acaciae*	MFLU 17-0496 ^T^	NR_160335	NG_069453	KY768870	-	-
*Phaeosphaeria acaciae*	KUMCC 20-0214	MW078431	MW078444	MW078482	MW082602	MW192765
*Phaeosphaeria ampeli*	MFLUCC 18-1641 ^T^	NR_165910	MK503808	MK503814	MK503802	-
*Phaeosphaeria ampeli*	MFLUCC 19-0150	MK503798	MK503809	MK503815	MK503803	-
*Phaeosphaeria avenaria f. sp. tritici*	CBS 289.52	MH857041	MH868572	-	-	KY090671
*Phaeosphaeria calamicola*	MFLUCC 14-1168 ^T^	KY511429	KY511423	KY511426	-	-
*Phaeosphaeria caricicola*	CBS 603.86	KF251182	GQ387590	GQ387529	-	KF252189
*Phaeosphaeria caricis-sectae*	CBS 146823 ^T^	NR_173034	MZ064470	-	-	MZ078195
***Phaeosphaeria chengduensis***	**KUNCC 23-13570**	**OR206391**	**OR206410**	**OR206400**	**OR195707**	**OR195716**
***Phaeosphaeria chengduensis***	**KUNCC 23-13571 ^T^**	**OR206392**	**OR206411**	**OR206401**	**OR195708**	**OR195717**
*Phaeosphaeria chiangraina*	MFLUCC 13-0231 ^T^	NR_155643	NG_069237	KM434289	KM434298	KM434307
*Phaeosphaeria chinensis*	MFLUCC 19-0217 ^T^	MN173212	MN173208	MN173216	-	-
*Phaeosphaeria chinensis*	KUMCC 19-0161	MN173213	MN173210	MN173217	-	-
*Phaeosphaeria cycadis*	KUMCC 18-0161 ^T^	NR_164445	NG_070078	NG_067700	MK359069	-
*Phaeosphaeria cycadis*	KUMCC 18-0162	MK356379	MK356353	MK356367	MK359070	-
*Phaeosphaeria elongata*	CBS 120250	MH863080	GU456327	GU456306	GU456261	GU456345
*Phaeosphaeria eustoma*	CBS 724.92	MH862385	JX681112	-	-	-
*Phaeosphaeria eustoma*	CBS 307.71	MH860138	JX681111	-	-	-
*Phaeosphaeria glyceriae-plicatae*	CBS 101261	MH862724	MH874330	-	-	-
*Phaeosphaeria juncophila*	CBS 575.86	AF439488	GU456328	GU456307	GU456283	-
*Phaeosphaeria luctuosa*	CBS 308.79	MH861209	GU301861	-	GU349004	KY090678
*Phaeosphaeria musae*	MFLUCC 17-2648	MK503796	MK503807	MK503813	-	-
*Phaeosphaeria musae*	CBS 120026	DQ885894	GU301862	GU296186	GU349037	GU357748
*Phaeosphaeria musae*	MFLUCC 11-0151	KM434268	KM434278	KM434288	KM434297	-
*Phaeosphaeria musae*	MFLUCC 11-0133	KM434267	KM434277	KM434287	KM434296	-
*Phaeosphaeria nodulispora*	URM 7220	KR092904	KR092903	-	-	-
*Phaeosphaeria norfolcia*	CBS 593.86	MH861997	MH873686	-	-	-
*Phaeosphaeria oryzae*	CBS 110110 ^T^	NR_156557	NG_069025	NG_061080	-	KF252193
*Phaeosphaeria oryzae*	MFLUCC 11-0170	KM434269	KM434279	-	-	KM434306
*Phaeosphaeria papayae*	S528	KF251187	KF251690	-	-	-
*Phaeosphaeria papayae*	CBS 135416	MH866082	MH877574	-	-	-
*Phaeosphaeria parvula*	CBS 605.86	MH862001	MH873689	-	-	-
*Phaeosphaeria parvula*	CBS 260.49	MH856516	MH868046	-	-	-
*Phaeosphaeria penniseti*	FU31020	MK503819	MK503825	MK503831	-	-
*Phaeosphaeria phoenicicola*	CPC 28711 ^T^	NR_156608	-	-	-	-
*Phaeosphaeria poagena*	CBS 136771 ^T^	NR_168146	NG_068518	-	-	-
***Phaeosphaeria poagena***	**KUNCC 23-13572**	**OR206393**	**OR206412**	**OR206402**	**OR195709**	**OR195718**
***Phaeosphaeria poagena***	**KUNCC 23-13573**	**OR206394**	**OR206413**	**OR206403**	**OR195710**	**OR195719**
*Phaeosphaeria podocarpi*	CBS 138903 ^T^	NR_137933	NG_070060	-	-	-
***Phaeosphaeria sichuanensis***	**KUNCC 23-13568**	**OR206389**	**OR206408**	**OR206398**	**OR195705**	**OR195714**
***Phaeosphaeria sichuanensis***	**KUNCC 23-13569 ^T^**	**OR206390**	**OR206409**	**OR206399**	**OR195706**	**OR195715**
*Phaeosphaeria sinensis*	MFLUCC 18-1552 ^T^	NR_163350	NG_070076	NG_065788	MK360072	-
*Phaeosphaeria sinensis*	KUMCC 17-0195	OM212456	OL813496	OL824792	ON203111	-
*Phaeosphaeria sinensis*	NCYUCC 19-0369	MN937237	MN937219	-	-	-
*Phaeosphaeria thysanolaenicola*	MFLUCC 10-0563 ^T^	NR_155642	NG_069236	KM434286	KM434295	KM434303
*Phaeosphaeriopsis aloes*	HKAS 115783	MZ493305	MZ493319	MZ493291	MZ508414	-
*Phaeosphaeriopsis beaucarneae*	MFLU 18-2586 ^T^	NR_170822	MT321813	MT321806	MT328756	-
*Phaeosphaeriopsis beaucarneae*	MFLU 18-2587	MT321800	MT321814	MT321807	MT328757	-
*Phaeosphaeriopsis glaucopunctata*	MFLUCC 13-0265	KJ522473	KJ522477	KJ522481	MG520918	-
*Phaeosphaeriopsis triseptata*	MFLUCC 13-0271	KJ522475	KJ522479	KJ522484	MG520919	KJ522485
*Phaeosphaeriopsis yuccae*	MFLUCC 16-0558	KY554482	KY554481	KY554480	MG520920	-
*Piniphoma wesendahlina*	CBS 145032 ^T^	NR_163375	NG_070464	-	-	MK442676
*Populocrescentia ammophilae*	MFLUCC 17-0665 ^T^	NR_157535	NG_059875	NG_063690	MG829231	-
*Populocrescentia forlicesenensis*	MFLUCC 14-0651 ^T^	NR_154326	KT306952	KT306955	MG520925	-
*Populocrescentia rosae*	TASM 6125 ^T^	-	MG829060	NG_062440	MG829232	-
*Pseudoophiobolus galii*	MFLUCC 17-2257 ^T^	MG520947	NG_069541	NG_063667	MG520926	-
*Pseudoophiobolus italicus*	MFLUCC 17-2255 ^T^	NR_156683	NG_059858	NG_063668	MG520927	-
*Pseudoophiobolus mathieui*	MFLUCC 17-1785	MG520951	MG520971	MG520992	MG520929	-
*Pseudoophiobolus urticicola*	KUMCC 17-0168 ^T^	NR_156686	NG_069543	NG_065140	MG520933	-
*Pseudoophiosphaerella huishuiensis*	HS13	MK522509	MK522499	MK522505	MK523389	-
*Pseudoophiosphaerella huishuiensis*	MFLUCC 17-1453	MN994590	MN994567	MN994613	MN998171	-
*Pseudoophiosphaerella huishuiensis*	MFLUCC 17-1463	MN994591	MN994568	MN994614	MN998172	-
*Pseudophaeosphaeria rubi*	MFLUCC 14-0259 ^T^	NR_154351	NG_067543	NG_061255	MG520934	-
*Pseudostaurosphaeria chromolaenae*	MFLUCC 17-1490 ^T^	NR_168854	NG_068696	NG_070147	MN998174	-
*Pseudostaurosphaeria chromolaenicola*	MFLUCC 17-1491	MN994594	MN994571	MN994617	MN998175	-
*Pseudostaurosphaeria chromolaenicola*	MFLUCC 17-1497 ^T^	NR_168855	NG_068697	NG_070148	MN998176	-
*Quixadomyces hongheensis*	KUMCC 20-0215 ^T^	NR_172441	MW264194	NG_074964	MW256816	MW269529
*Quixadomyces hongheensis*	HKAS112346	MW541826	MW541822	MW541833	MW556134	MW556136
*Sclerostagonospora cycadis*	CBS 123538 ^T^	NR_160231	FJ372410	-	-	-
*Sclerostagonospora elegiae*	CBS 118142 ^T^	NR_176098	NG_081272	-	-	-
*Sclerostagonospora ericae*	CBS 141318 ^T^	NR_145199	NG_070625	-	-	-
*Sclerostagonospora fusiformis*	CBS 118152	JX517283	JX517292	-	-	-
*Sclerostagonospora lathyri*	MFLUCC 14-0958 ^T^	NR_158956	NG_069566	NG_063692	MG829235	-
*Sclerostagonospora pinguis*	CBS 118146 ^T^	NR_176097	NG_081271	-	-	-
*Sclerostagonospora rosae*	MFLU 18-0115 ^T^	NR_157541	NG_069567	NG_065151	MG829236	-
*Sclerostagonospora rosicola*	MFLUCC 15-0129 ^T^	MG828957	MG829068	NG_063693	MG829237	-
*Scolicosporium minkeviciusii*	MFLUCC 12-0089	-	KF366382	KF366383	-	-
*Septoriella agrostina*	MFLU 18-0113 ^T^	NR_157533	NG_069561	NG_062198	MG829227	-
*Septoriella allojunci*	MFLU 15-0701 ^T^	KU058718	KU058728	NG_065141	MG520935	-
*Septoriella ammophilicola*	MFLU 17-2571 ^T^	MN047087	NG_070468	NG_068403	MN077065	-
*Septoriella ammophilicola*	MFLU 17-2568	MN047088	MN017848	MN017914	MN077066	-
*Septoriella artemisiae*	MFLUCC 17-0693 ^T^	MG828929	MG829038	NG_063688	-	-
*Septoriella arundinicola*	MFLU 16-0225 ^T^	MG828946	MG829056	NG_062199	MG829228	MG829261
*Septoriella arundinis*	MFLUCC 15-0702 ^T^	KU058716	KU058726	NG_061283	MG520921	-
*Septoriella asparagicola*	MFLUCC 16-0379 ^T^	NR_165908	NG_070081	NG_067708	MK443385	MK443387
*Septoriella bromi*	MFLUCC 13-0739 ^T^	KU058717	KU058727	-	-	-
*Septoriella callistemonis*	CBS 146822 ^T^	NR_173033	MZ064469	-	-	MZ078194
*Septoriella camporesii*	KUMCC 16-0113 ^T^	NR_168233	MN648201	NG_068422	-	-
*Septoriella chlamydospora*	MFLUCC 15-0177 ^T^	NR_154508	KU163654	NG_063595	-	-
*Septoriella dactylidicola*	MFLUCC 14-0002 ^T^	-	KY657264	KY657265	-	-
*Septoriella dactylidis*	MFLU 15-2720 ^T^	NR_154507	KU163656	-	-	-
*Septoriella elongata*	HA 4444	KM491546	KM491548	KM491549	-	-
*Septoriella festucae*	MFLUCC 17-0056 ^T^	KY824766	KY824767	KY824769	-	KY824768
*Septoriella forlicesenica*	MFLUCC 15-0470	KX926422	KX910095	KX950406	MG520922	KY131966
*Septoriella garethjonesii*	MFLUCC 15-0469	KX926425	KX954390	KY205717	MG520923	KX898363
*Septoriella garethjonesii*	MFLUCC 13-0275	KX926420	KX910093	KX950405	-	KX880500
*Septoriella germanica*	CBS 145372 ^T^	NR_164459	MK540035	-	-	MK540096
*Septoriella hibernica*	CBS 145371 ^T^	NR_164460	MK540036	-	-	MK540097
*Septoriella hirta*	CBS 536.77 ^T^	NR_145192	KR873278	-	-	KR873324
*Septoriella hollandica*	CBS 145374 ^T^	NR_164461	MK540037	-	-	MK540098
*Septoriella hubertusii*	CBS 338.86 ^T^	NR_155786	KF251733	-	-	KF252235
*Septoriella italica*	MFLUCC 13-0267	KX926421	KX910094	KX950409	MG520924	KX891169
*Septoriella leuchtmannii*	CBS 459.84 ^T^	NR_163526	NG_057999	KY090700	-	KF252195
*Septoriella luzulae*	MFLUCC 14-0826 ^T^	NR_154121	NG_069310	NG_063585	-	-
*Septoriella muriformis*	MFLUCC 13-0277	KX926415	KX910089	KX950400	-	KX863710
*Septoriella neoarundinis*	MFLUCC 15-0046	KY706140	KY706130	KY706135	KY706144	KY706147
*Septoriella neoarundinis*	MFLUCC 15-0027 ^T^	NR_154541	KY706129	NG_063636	MG520936	-
*Septoriella neodactylidis*	MFLUCC 14-0966 ^T^	NR_157511	NG_069554	NG_061288	MG829199	MG829253
*Septoriella neodactylidis*	MFLUCC 13-0618	KP744432	KP744473	KP753946	-	-
*Septoriella neomuriformis*	MFLUCC 17-0372 ^T^	MF611637	MF611638	MF611639	-	-
*Septoriella oudemansii*	CBS 138012 ^T^	KR873250	KJ869224	-	-	-
*Septoriella paradactylidis*	KUMCC 18-0154	MK356371	MK356345	MK356359	-	-
*Septoriella paradactylidis*	MFLU 17-0498 ^T^	NR_164251	NG_070411	-	-	-
***Septoriella paradactylidis***	**KUNCC 23-14219**	**OR206395**	**OR206414**	**OR206404**	**OR195711**	**OR195720**
*Septoriella phragmitis*	CPC 24118 ^T^	NR_132926	NG_069285	-	-	-
*Septoriella poae*	CBS 136766 ^T^	NR_155793	NG_067494	-	-	-
*Septoriella pseudophragmitis*	CBS 145417 ^T^	NR_164468	MK560160	-	-	MK559450
*Septoriella rosae*	MFLU 18-0114 ^T^	NR_157534	NG_069562	NG_065150	MG829230	-
*Septoriella shoemakeri*	MFLUCC 14-0963 ^T^	NR_157512	MG829003	NG_063678	MG829200	-
*Septoriella subcylindrospora*	MFLUCC 13-0380 ^T^	KT314184	KT314183	KT314185	-	-
*Septoriella tridentina*	MFLUCC 15-0474 ^T^	NR_165849	NG_069389	NG_068369	-	KX891170
*Septoriella vagans*	CBS 604.86	KF251193	KF251696	-	-	KF252200
*Septoriella verrucispora*	MFLUCC 19-0163 ^T^	MK522508	MK522498	MK522504	MK523388	-
*Setomelanomma holmii*	CBS 110217	KT389542	GU301871	GU296196	GU349028	GU371800
*Setophoma caverna*	R150	MK511944	MK511965	-	-	-
*Setophoma sacchari*	CBS 333.39 ^T^	NR_145173	NG_057837	NG_062779	-	KF252250
*Setophoma sacchari*	MFLUCC 12-0241	KJ476145	KJ476147	KJ476149	KJ461318	-
*Setophoma vernoniae*	CBS 137988 ^T^	NR_168153	KJ869198	-	-	-
*Stagonospora foliicola*	CBS 110111	KF251256	KF251759	EU754118	-	-
*Sulcispora pleurospora*	CBS 460.84	AF439498	-	-	-	-
*Sulcispora supratumida*	MFLUCC 14-0995 ^T^	NR_160325	NG_067530	NG_065627	MH665366	-
*Tintelnotia destructans*	CBS 127737 ^T^	NR_147684	NG_058274	NG_063077	-	KY090683
*Tintelnotia opuntiae*	CBS 376.91 ^T^	NR_147683	NG_067267	NG_062790	-	KY090680
*Vittaliana mangrovei*	NFCCI 4251 ^T^	NR_165870	NG_067809	NG_067664	MG767314	MG767315
*Vrystaatia aloeicola*	CBS 135107	KF251278	KF251781	-	-	KF252283
*Wingfieldomyces cyperi*	CBS 141450 ^T^	NR_155805	NG_059684	-	-	MK540101
*Wojnowiciella cissampeli*	CPC 27455 ^T^	NR_155972	NG_069358	-	LT990616	-
*Wojnowiciella clematidis*	MFLUCC 17-2159 ^T^	NR_170812	MT214582	MT226695	MT394641	MT394698
*Wojnowiciella eucalypti*	CPC 25024 ^T^	NR_137996	NG_070629	-	LT990617	-
*Wojnowiciella kunmingensis*	KUMCC 18-0159 ^T^	NR_164446	NG_070079	NG_067701	MK359071	MK359078
*Wojnowiciella leptocarpi*	CBS 115684 ^T^	NR_155973	KX306800	-	LT990615	LT990646
*Wojnowiciella spartii*	MFLUCC 13-0402 ^T^	KU058719	KU058729	NG_063670	MG520937	-
*Xenophaeosphaeria grewiae*	CBS 138867 ^T^	NR_137944	NG_058124	-	-	-
*Xenophoma puncteliae*	CBS 128022	JQ238617	JQ238619	-	-	-
*Xenoseptoria neosaccardoi*	CBS 120.43	KF251280	KF251783	-	-	KF252285
*Xenoseptoria neosaccardoi*	CBS 128665	KF251281	KF251784	-	-	KF252286
*Yunnanensis chromolaenae*	MFLUCC 17-1486	MN994596	MN994573	MN994619	MN998177	-
*Yunnanensis chromolaenae*	MFLUCC 17-1487 ^T^	NR_168856	NG_068698	NG_070149	MN998178	-
*Yunnanensis phragmitis*	MFLUCC 17-0315 ^T^	MF684862	MF684863	MF684867	MF683624	-

## Data Availability

The datasets generated for this study can be found in the NCBI GenBank and MycoBank.
